# Geochemical and isotopic tracers to define the aquifer’s vulnerability: the case study of the alluvial multi-aquifer system of the Friulian plain

**DOI:** 10.1007/s10661-023-11359-7

**Published:** 2023-05-31

**Authors:** Dino Di Renzo, Antonietta Rizzo, Chiara Telloli, Stefano Salvi, Elena Marrocchino, Daniel Nieto Yàbar, Carmela Vaccaro

**Affiliations:** 1 Department of Physics and Earth Sciences, University of Ferrara, Via Saragat 1, 44122 Ferrara, Italy; 2 Italian National Agency for New Technologies Energy and Sustainable Economic Development - Fusion and Technology for Nuclear Safety and Security Department - Nuclear Safety, Security and Sustainability Division - Via Martiri Di Monte Sole 4, FSN-SICNUC-TNMT, 40129 Bologna, Italy; 3 Department of Environmental and Prevention Science, University of Ferrara, C.So Ercole I D’Este 32, 4412 Ferrara, Italy; 4 OGS National Institute of Oceanography and Applied Geophysics, Borgo Grotta Gigante, 42/C, 34010 Trieste, Italy

**Keywords:** Groundwater recharge, Tritium measurement, Isotope tracers, Vulnerability of water bodies

## Abstract

The Friuli-Venezia Giulia Region (north of Italy) is characterized by the presence of high-quality freshwater resources which benefit local citizens, animals, environmental habitats, and also agriculture and production activities. Waters from wells, canal, and wastewater selected in the Fiume Veneto area, through a detailed lithological modeling, were sampled and analyzed to characterize them from a geochemical point of view. The chemical and isotopic characterization made it possible to establish provenance, and the average age of water used, making available the estimation of the relationships between recharge capacity and water use in the Fiume Vento area. The focus of this study is to define the average age of the resources based on the time required for the recharge contributions to compensate the losses induced by exploitation. The results made it possible to support the plans for a water balance using the provenance and average age of water sources for the protection of water reserves formed by the multi-aquifer system of the high and medium Friuli plain. The methodology applied has followed the legislation of the water directive considering the overexploitation due to unauthorized withdrawals of the sampling area.

## Introduction

The safeguarding of surface and underground water resources is one of the priorities of European policies and of the Italian government (EU Water Framework Directive, 2000). Geochemistry and in particular the use of environmental isotopes provide useful markers to define the relationships between surface and groundwater and to date the recharge times (Frei et al., 2020; Rashid et al., 2019). The monitoring of the geochemical and isotopic response to seasonal fluctuations induced by climatic conditions and the exploitation of water bodies allows the formulation of hydrodynamic models for the sustainable use of the water resource (Beal et al., 2019).

Water availability, in quality and quantity, is essential for the supply of drinking water, to support safe agriculture and to ensure the necessary resources for human activities in order to prevent dynamics of competitive use (Krueger et al., 2019; Mercure et al., 2019). Unfortunately, economic interests can often prevail over the protection of aquifers, causing damage to human health, and food safety. Groundwater over-exploitation is often produced by the lack of awareness of the dynamics of recharging (Lili et al., 2020), and it is therefore essential to make people understand the impact that the uncontrolled use of the water resource implemented in the last century has caused an important waste of water resources dispersing water in the surface drainage network.

The Friuli-Venezia Giulia Region has high-quality freshwater resources which are associated with the well-being of citizens, the high presence of different kinds of habitats, and also the high-water availability for agriculture and production activities. In particular, the investigated area of Fiume Veneto is part of the mid-plain area of transition between the undifferentiated aquifer of the Upper Plain and the confined aquifer that characterizes the presence of resurgences (Maples et al., 2020). Today, the excessive water demand has highlighted the vulnerability of the aquifer system due to the state of exploitation. The over-exploitation of the aquifer with the dispersion of water in the surface hydraulic network (John et al., 2021) is increased by the high amount of water-well withdrawals (Yuanyuan et al., 2018) where water is uncontrollably released due to the absence of taps.

The geochemical characterization of freshwater in the Fiume Veneto area is part of a collaboration between Livenza Tagliamento Acque (LTA) and Ferrara University to know and preserve strategic drinking water resources from the quantitative and qualitative degradation induced by anthropogenic activities and by prolonged drought period during summer season (Adimalla & Venkatayogi, 2018; Sunitha & Reddy, 2022). This lack of knowledge on the vulnerability of the area is often not tangible due to the high recharge produced by precipitation (ARPA FVG, 2021). Every year, the phenomenon becomes concrete in summer when the variation of the rainfall regime and the increase in withdrawals for irrigation purposes bring out the depletion of the groundwater aquifer. This event causes the regression of the resurgence line and lack of water availability in the wells of the confined aquifer (Telloli et al., 2022). The confined aquifer, in fact, is the one that most records the depletion of resources due to the high presence of dispersing wells which makes losses unsustainable in periods of low recharge.

The knowledge of the isotopic composition (δ^2^H, δ^18^O, and ^3^H) in surface and groundwater in relation to the isotopic composition of the precipitations allows us to determine the average residence time of the water in the aquifers (Campbell et al., 2021; Krajcar Bronić & Barešić, 2021), the possible interconnections between different aquifers (Andries et al., 2021; Mahlangu et al., 2020), the origin of waters (Andries et al., 2021; Catalano et al, 2014), and the dynamics of processes in surface waters (Ramaroson et al., 2018; Schmidt et al., 2020). In addition, although the ^3^H concentration in the atmosphere in recent years has reached values in line with the environmental threshold, thanks to the electrolytic enrichment methodologies it is possible to determine the low concentrations of ^3^H using the liquid scintillation counting technique (Belachew et al., 2018; Lin et al., 2020).

For this reason, in this research work, a geochemistry and isotopic approach was adopted on waters collected from wells, canal, and wastewater which is useful for implementing geochemical knowledge and optimally defining and characterizing the interactions between aquifers and the surface waters (e.g., wastewater or canal water) and to estimate the relationship between recharge capacity and water use (Hao et al., 2019; Joshi et al., 2018). Specific markers were identified highlighting the differences between groundwater and other types of water using the dissolution kinetics of carbonates (rCa/rMg ratio and isotope ratio of δ^18^O and δ^2^H) (Jesiya et al., 2021; Li et al., 2019a) and the ^3^H isotope (Li et al., 2019b; Niu et al., 2020). These analyses permit defining the average age of the resources as the time required for the recharge contributions to compensate for the losses induced by exploitation and resurgence (Balocchi et al., 2022; Barbieri, 2019). Furthermore, these data allow estimating the critical values of depletion that can compromise the quality and quantity of the resource.

This multidisciplinary approach allows us to chemically characterize the collected waters and identify the type of water especially of the surface channels into which the domestic wells are not declared to the competent authorities’ discharge. All this information obtained at the sampling site made it possible to identify the type of aquifer from which most of the domestic wells draw and to understand the critical issues of groundwater over-exploitation. This is essential for the competent authorities to implement mitigation plans, especially for future periods of water scarcity due to climate change.

Thanks to this knowledge it is possible to plan the use of the resource following the water cycle in which the recharge allows to balance the uncontrolled withdrawals and therefore to preserve the aquifers from degradation and depletion phenomena (Li et al., 2018; Xiang et al., 2021).

## Materials and methods

### Study area

**Fig. 1:**
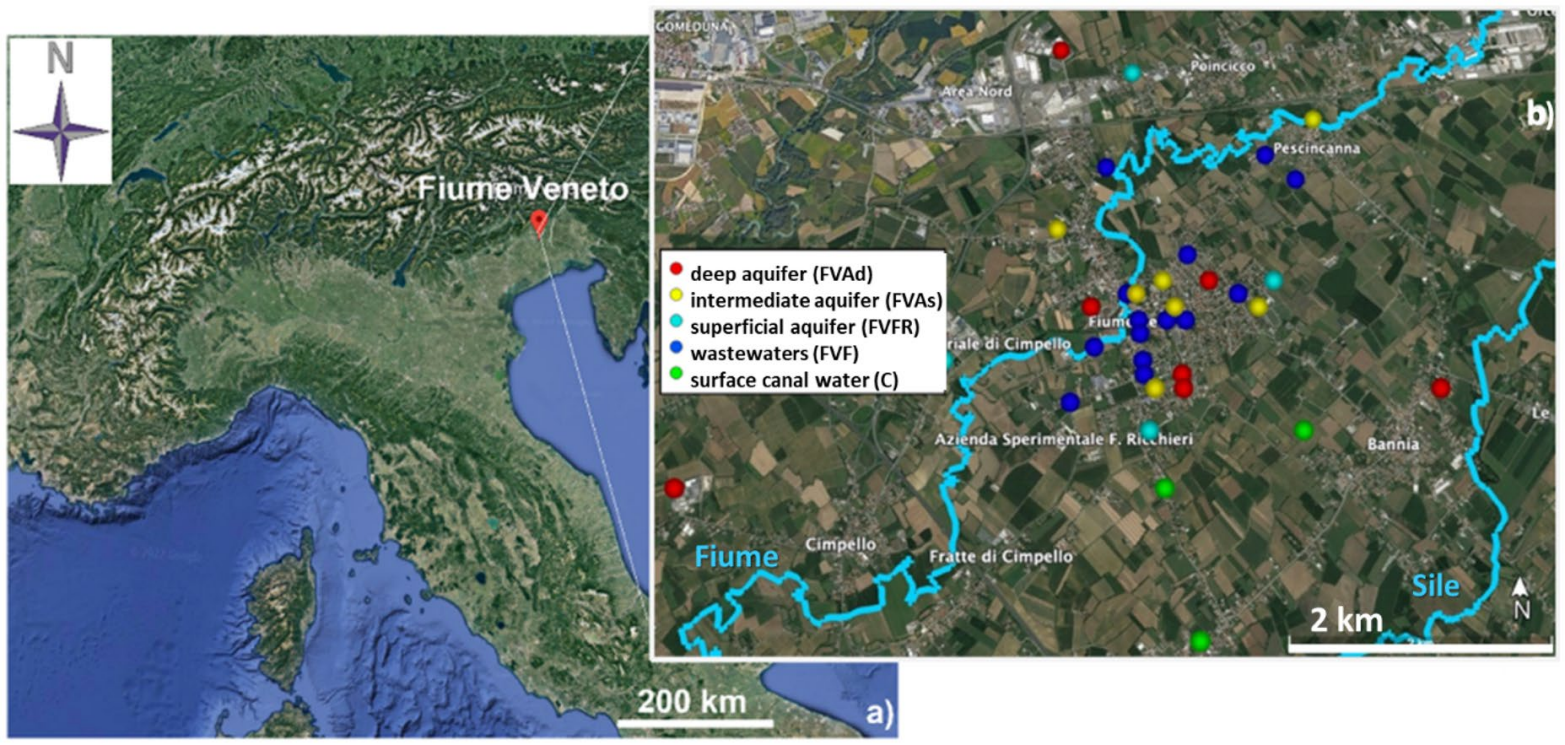
Map of the sampling site of Fiume Veneto obtained using Google Earth software Version 9.163.0.0: **a** Italian map in which colored in red the location of Fiume Veneto; **b** Fiume Veneto and all the territories and localities selected for the water sampling colored in light blue superficial aquifer water samples (FVFR), in yellow intermediate aquifer water samples (FVAs), in red deep aquifer water samples (FVAd), in blue wastewater samples (FVF), in green the surface canal water samples (C). Colored with light blue lines the two main Rivers Fiume and Sile

The studied area is located in the central-southern sector of the province of Pordenone, within the municipal area of Fiume Veneto (Fig. 1a), in the Lower Pordenone Plain downstream of the resurgence line and belonging to the hydrogeological basin of the Friuli Plain (Friuli-Venezia Giulia Region, northeast of Italy). The resurgence line is an important hydrologic element that divides the plain into two physiographic zones, the high plain characterized by a phreatic aquifer and the low plain that consists of several confined aquifer systems. The resurgence belt represents a kind of “overflow” for the high plain into the low plain.

The municipality of Fiume Veneto belongs to the low plain close to the resurgence line and has an extension of 35.8 km^2^. It is characterized by low altitudes ranging from a maximum of 30 m above sea level (a.s.l.) to a minimum of 14 m a.s.l., and it is mainly characterized by canals with very narrow floodplain areas.

The research study was extended to the towns of the municipality of Fiume Veneto into the territories of Bannia, Cimpello, Pescincanna, Praturlone, and the localities of Borgo Tavella, Fiume Piccolo, Marzinis, Piandipan, Rivatte, and Villanova (Fig. 1b).

#### Climatological context of the study area

The orographic and climatic conditions mean that the average annual temperature generally ranges between 12 and 14 °C (Autonomous Region of Friuli Venezia Giulia, 2018). Therefore, following the Köppen-Geiger climate classification (Peel et al., 2007), the climate of the studied area can be considered a moderate continental climate with a prevalence of hot-humid air flows from the southwest and fewer events of dry cold air flow from the northwest in winter. The recharge of the aquifers is favored by the flows of humid air coming from the south, which produce abundant rainfall (rain and snow) that characterize the spring and summer storms (Autonomous Region of Friuli Venezia Giulia, 2018).

In the past, this area has not suffered from water scarcity as it has a humid temperate climate regime, the average annual rainfall with values between 1000 and 2000 mm, and limited differences in rainfall between the rainy and dry months (ARPA FVG, 2021).

#### Geological setting

The municipality of Fiume Veneto, included in the geological map 086 “San Vito al Tagliamento” (Zanferrari et al., 2008), is part of the eastern sector of the Po valley consisting of the deposits of alpine rivers (Castiglioni, 1999). The plain is characterized by the presence of terrigenous sediments that are from the Tertiary to the Quaternary filled the tectonic morphological depressions linked to the impact of the south-verging thrust of the Alpine chain on the foreland (Doglioni, 1993). The sediments are related to the Cellina and Meduna rivers and to the tributaries of the Tagliamento River (Stefanini & Cucchi, 1978).

The sampling site is characterized by the Pliocene–Quaternary continental succession (Bartolini et al., 1996) with the presence of clayey-silty sediments that incorporate within them two permeable macro-levels characterized by gravelly and sandy sediments of the Po River, from about -20 to -50 m a.s.l. and from about − 140 to − 180 m a.s.l. (Zanferrari et al., 2008).

#### Hydrogeological setting

From the hydrographic point of view, the sampling site is crossed by two main watercourses (Fig. 1b, colored in light blue). The first is the Fiume River, which crosses Pescincanna town, Fiume Veneto, and Cimpello. The other is the Sile River, which flows along the south-eastern part of the territory crossing Bannia and Praturlone. Both rivers come from the same resurgence area. As regards the multi-aquifer system, in the more superficial layers, from 0 to − 4.0 m, there are aquifers fed mainly by recharged from rainfall, while deeper, there are artesian aquifers fed by the free aquifer of the high plain (Stefanini & Cucchi, 1977).

As far as the hydrogeology of the area is concerned, it should be noted that the most important element is represented by the water table (Yàbar et al., 2012) which, at the time of the investigations, was intercepted from a minimum of 1.30 m from the ground level to a maximum of 2.20 m from the countryside level. From previous studies, carried out in the area covered by the present study, the level of the surface water table can usually be found at a depth between 0.80 and 2.50 m from the ground level (Fusetti, 2016).

### Lithologic modeling of the aquifer system in the Fiume Veneto area

The lithologic modeling is intended to characterize the complexity of the hydro-lithographic framework of the study area and to show the spatial trends and variability of thickness and heterogeneity of the aquifer system. Relevant information for the identification of optimal sampling points for this study: 93 well log data were used, on an area of approximately 53.68 km^2^, for building the lithologic model. The first step was to standardize the log data according to the main lithological classes: gravel, gravel with sand, sand, sand with clay, clay, and clay with sand. This data subsequently was processed using lithologic modeling techniques based on the “solid modeling” concept provided in the RockWorks 2021 software package, in which a true three-dimensional gridding process was used. A “box” was created of regularly spaced nodes from irregularly spaced data by interpolating measured values of lithology types. The resolution of the model was 100 m (X) × 100 m (Y) × 2 m (Z). The resulting discretization consisted of 79 X nodes × 56 Y nodes × 107 Z nodes, thus having 473,368 solid model nodes. In the present study, the method of inverse distance is used for interpolation of the available lithologic well log data.

The results obtained from the lithological modeling were represented by conventional 2D lithologic sections which showed a good direct correlation between the log well data.

### Sampling methodology

The sampling campaign was conducted in June 2021 in collaboration with the LTA consortium “Livenza Tagliamento Acque,” ENEA and the University of Ferrara to investigate the geochemistry of the water samples and to determine the type of water especially collected on private wells and on superficial canals. To obtain a reliable estimate of the relationship between recharge capacity and water consumption in the Fiume Veneto area, it was decided to carry out a geochemical and isotopic characterization of the waters deriving from a potential aquifer identified with the lithological model.

During the sampling campaign, a total of 36 water samples were collected (Fig. 1b) at a depth of approximately − 200 m a.s.l., knowing that all the surface and groundwater flow directions mainly are from N to S (Zini et al., 2013). Both private water wells and water wells managed by LTA were investigated and sampled. The water samples were divided into:

- 4 samples characterizing water from a superficial aquifer collected at a depth of about − 3.5 m (FVFR, colored in light blue in the map of Fig. 1b);
- 7 samples characterizing water from the intermediate aquifer collected at a depth of about − 30/80 m to the ground (FVAs, colored in yellow in the map of Fig. 1b);
- 7 samples characterizing water from deep aquifer collected at a depth of about − 180 m to the ground (FVAd, colored in red in the map of Fig. 1b);
- 15 samples characterizing the wastewater collected from the sewerage network (FVF, colored in blue in the map of Fig. 1b);
- 3 samples representing waters of two surface canals (C, colored in green in the map of Fig. 1b) connecting to the Sile River.

The sampling campaign was divided into two parts:

- In situ: measuring the main chemical/physical parameters (temperature “T,” dissolved oxygen “DO,” pH, electrical conductivity “EC”) using a multiparametric probe Hanna Instruments (HI9828 model), performing the alkalinity test using the blue bromophenol indicator and acid solution through the measuring kit Hanna Instruments (HI3811 model) and samples collection.
- In the laboratory: chemical analyses through ion chromatography (IC), inductively coupled plasma mass spectrometry (ICP-MS), cavity ring down spectroscopy (CRDS), and ^3^H analysis.

The sampling method was differentiated according to the type of water. For the water sampled in the drainage networks, the sampling was carried out instantly while for the groundwater the samples were collected by means of an electric pump after having let the water flow out for about 5 min.

Furthermore, different types of sampling were performed depending on the analyses. Water samples were collected in 100 ml polyethylene containers with a safety cap and screw cap for the analysis of stable isotopes (δ^2^H and δ^18^O); in addition, water samples previously filtered with a 0.45-µm cellulose acetate filter were collected in 50-ml polyethylene containers with safety cap and screw cap for the cations and anions analysis.

The water samples for ^3^H analyses were, on the other hand, collected in 1-l amber glass containers to avoid, or at least limit, photoluminescence effects and possible degradation of the sample due to photochemical causes. The samples were stored in a closed environment without color and light sources until the analysis.

### Analytical techniques

In all the wells, the main parameters required by the EU Water Framework Directive (2000) must be analyzed directly on-site (Table 1). In addition, chemical analyses were carried out in the Laboratory of the Department of Physics and Earth Sciences of the University of Ferrara (Emilia Romagna region, north-east of Italy) according to standard methods (APHA, 1992):

- Anions (Cl^−^, NO_3_^−^, and SO_4_^2−^) were analyzed by an ICS-1000 Ion Chromatography System (DIONEX, Sunnyvale, CA, USA). Detection limits were 0.03 mg/L for Cl^−^ and SO_4_^2−^, and 0.01 mg/L for NO_3_;
- Cations (Na^+^, Mg^2+^, K^+^, and Ca^2+^) analyzed by an ICP-MS Thermo Series X-I spectrometer (Thermo Fischer Scientific, Waltham, MA, USA). The water samples were diluted with 2% HNO_3_^−^ to stabilize the sample and prevent its precipitation and adsorption phenomena on the internal surfaces of vessels and pipes. Detection limits were 1 mg/L for Na^+^, Mg^2+^, Ca^2+^, and HCO_3_^−^, and 0.1 mg/L for K^+^

**Table 1:** Main chemical and physical parameters: “T” temperature, “DO” dissolved oxygen “DO,” pH, electrical conductivity “EC”.

ID	Depth	T	DO	pH	EC	Na^+^	Mg^2+^	K^+^	Ca^2+^	Cl^−^	NO_3_^−^	SO_4_^2−^	HCO^3−^	δ^2^H	δ^18^O	^3^H	^3^H std dev
	(m)	(°C)	(mg/L)		(μS/cm)	(mg/L)	(mg/L)	(mg/L)	(mg/L)	(mg/L)	(mg/L)	(mg/L)	(mg/L)	(‰)	(‰)	(TU)	(± TU)
FVFR_1	3.5	16.1	1.4	8.6	483.0	8.0	25.9	2.0	85.9	6.9	8.1	38.9	210.0	− 46.3	− 8.6	5.2	0.5
FVFR_2	3.5	16.1	1.3	8.1	483.0	5.4	38.4	2.7	132.8	2.6	5.7	8.3	420.0	− 35.5	− 6.3	5.9	0.5
FVFR_3	3.5	15.9	0.7	8.2	338.0	4.1	14.5	4.7	68.0	2.7	0.4	7.4	255.0	− 20.6	− 3.7	7.2	0.6
FVFR_4	3.5	15.7	2.2	8.3	292.0	3.3	16.1	10.2	70.1	4.0	1.3	25.2	153.0	− 29.0	− 5.4	6.8	0.6
FVAs_1	30.0	13.3	4.5	8.8	353.0	2.2	26.1	0.4	60.1	2.2	8.2	46.8	156.0	− 51.2	− 8.3	2.7	0.2
FVAs_2	33.0	13.2	4.2	8.7	348.0	2.0	23.0	0.4	55.5	2.1	5.8	66.7	150.0	− 54.0	− 9.5	3.4	0.3
FVAs_3	80.0	16.2	4.3	8.8	377.0	2.7	25.9	0.5	66.2	2.1	5.1	86.1	150.0	− 54.3	− 9.6	2.9	0.3
FVAs_4	30.0	15.6	5.2	9.0	321.0	1.8	26.9	0.5	55.2	1.9	4.4	71.6	126.0	− 52.1	− 9.0	3.9	0.3
FVAs_5	30.0	13.6	4.6	8.8	371.0	2.3	25.6	0.4	59.9	2.1	5.2	69.7	144.0	− 53.8	− 9.2	3.6	0.3
FVAs_6	80.0	13.2	4.6	8.8	361.0	2.5	28.3	0.5	66.9	2.1	6.5	55.8	171.0	− 53.4	− 8.8	4.1	0.4
FVAs_7	80.0	13.5	4.7	8.7	458.0	1.5	24.9	0.4	52.6	2.0	5.5	75.0	135.0	− 53.6	− 8.7	4.2	0.4
FVAd_1	194.0	20.0	0.5	7.2	350.0	1.3	18.3	0.3	38.8	1.7	5.9	21.6	153.0	− 53.2	− 8.3	2.4	0.2
FVAd_2	140.0	22.7	3.9	9.1	360.0	2.1	30.1	0.5	63.8	1.9	4.1	94.7	144.0	− 55.7	− 9.4	3.0	0.3
FVAd_3	170.0	14.0	5.6	8.4	337.0	1.7	24.5	0.3	50.5	1.9	3.9	94.1	135.0	− 54.9	− 9.6	3.3	0.3
FVAd_4	185.0	13.4	5.3	8.7	339.0	2.2	24.8	0.4	59.0	2.1	6.4	56.8	156.0	− 55.2	− 8.9	2.1	0.2
FVAd_5	150.0	13.1	4.0	8.9	250.0	1.3	23.5	0.3	48.6	1.8	6.9	27.4	123.0	− 54.9	− 8.1	4.1	0.4
FVAd_6	174.0	14.6	5.5	8.4	365.0	2.0	32.2	0.5	67.4	1.9	3.2	118.6	144.0	− 58.8	− 8.9	3.7	0.3
FVAd_7	180.0	13.8	5.0	8.7	295.0	1.6	24.3	0.4	49.8	1.7	3.8	59.4	135.0	− 57.4	− 8.9	4.2	0.4
FVF_1	-	14.7	3.8	8.1	314.0	1.3	14.0	0.4	34.3	2.5	8.2	40.1	168.0	− 55.2	− 9.0	4.0	0.4
FVF_2	-	14.5	5.1	8.5	426.0	1.5	14.0	0.5	46.4	3.0	6.7	61.2	150.0	− 54.1	− 9.0	4.3	0.4
FVF_3	-	14.7	5.5	8.8	382.0	1.8	13.0	0.3	33.6	2.5	5.5	74.5	180.0	− 54.0	− 8.9	3.6	0.3
FVF_5	-	15.3	4.9	7.5	353.0	1.5	13.5	0.5	39.8	3.1	6.3	66.1	180.0	− 54.5	− 9.1	5.0	0.4
FVF_6	-	14.9	5.4	8.7	410.0	4.3	12.6	0.5	33.1	11.2	6.0	69.4	174.0	− 56.7	9.3	3.4	0.3
FVF_7	-	15.4	4.6	8.6	405.0	2.4	14.2	0.5	36.9	4.4	5.7	73.7	195.0	− 56.7	− 9.4	4.0	0.4
FVF_8	-	15.8	4.8	8.6	367.0	2.9	13.5	0.5	35.5	6.1	5.2	76.1	180.0	− 53.6	− 9.0	3.8	0.3
FVF_9	-	15.4	4.3	8.6	404.0	2.0	13.2	0.7	36.1	3.7	5.3	74.0	180.0	− 51.0	− 8.8	4.1	0.4
FVF_10	-	15.7	4.7	8.5	425.0	2.0	14.7	0.5	43.4	3.2	3.7	91.4	195.0	− 52.5	− 8.7	3.5	0.3
FVF_11	-	17.5	3.7	8.7	373.0	1.8	12.9	0.5	32.1	2.4	4.6	73.1	210.0	− 55.3	− 8.4	4.0	0.3
FVF_12	-	15.2	6.0	8.3	377.0	1.9	17.3	0.5	38.3	2.6	4.7	86.4	150.0	− 56.0	− 8.6	3.5	0.3
FVF_13	-	15.0	4.7	7.7	386.0	2.0	16.7	0.6	38.4	2.8	5.1	77.2	180.0	− 55.9	− 8.9	4.0	0.3
FVF_14	-	15.2	4.7	8.7	344.0	1.9	13.8	0.5	29.0	3.1	4.7	74.9	129.0	− 53.3	− 9.5	3.4	0.3
FVF_15	-	16.0	4.7	8.7	346.0	1.0	12.3	0.3	28.5	1.9	4.1	77.9	135.0	− 57.7	− 9.0	3.6	0.3
FVF_16	-	15.8	6.0	8.7	346.0	1.7	14.0	0.5	29.3	2.7	4.9	72.1	150.0	− 56.7	− 9.1	3.1	0.3
C_19	-	22.2	4.6	9.1	454.0	1.8	16.6	0.6	45.6	3.4	3.2	84.4	216.0	− 51.3	− 8.5	5.0	0.4
C_23	-	16.8	2.0	9.3	315.0	2.1	17.2	0.4	35.5	3.0	3.8	84.3	147.0	− 53.0	− 9.1	5.2	0.4
C_29	-	21.8	4.0	9.6	387.0	1.6	16.7	0.3	40.2	3.0	5.4	89.5	150.0	− 50.0	− 8.8	3.4	0.3

FVFR means superficial aquifer water samples, FVAs means intermediate aquifer water samples, FVAd means deep aquifer water samples, FVF means wastewater samples, and C means the surface canal water samples.

Stable isotopic analyses were carried out on the same water samples using the CRDS LWIA 24-d isotopic analyzer (Los Gatos Research Inc.) (Telloli et al., 2022). Analytical precision and accuracy, based on replicate analyses of standards, were better than 0.07‰ and 0.2‰ for δ^18^O and δ^2^H, respectively.

Finally, the ^3^H level in the water samples was determined at the Environmental Radiometry Laboratory (FSN-SICNUC-TNMT) of the ENEA Research Centre of Brasimone (Emilia-Romagna region, north-east of Italy) using the liquid scintillation counting (LSC) technique by a Quantulus™ 1220 low-background counter (PerkinElmer, USA), as described in the ISO 9698 (2019). All the samples were previously subject to an electrolytic enrichment process prior to measurement, as described in Telloli et al. (2022), because for the aquifer bodies investigated, the recharge times were rather long, and therefore, ^3^H values could be below the detectability threshold for most of the sampled areas. The average analytical accuracy for ^3^H was estimated at 0.5 TU (tritium unit).

### Multivariate statistical analysis (MSA)

The data was treated by means of an MSA following the CoDa approach proposed by Blake et al. (2016). The CoDa approach has been widely used in soil geochemistry studies (Buccianti et al., 2018; Carranza, 2011; Reimann and Caritat, 2012) and hydrogeological studies (Blake et al., 2016; Bondu et al., 2020; Herms et al., 2021; Taussi et al., 2022). In this technique, data are fully considered, enhancing their relative multivariate behavior in the correct sample space (Buccianti et al., 2015), and the technique has been developed based on the concepts proposed by Aitchison (1982). It is critically important that the compositional nature of environmental data be taken into consideration for practically any aspect of statistical data analysis (Filzmoser et al., 2009), as a failure to do so has been shown to generate misleading results (e.g., Otero et al., 2005; Wang et al., 2014). Compositional data can be treated prior to statistical analysis by using a family of log-ratio transforms (Aitchison, 1986; Egozcue et al., 2003) to convert the original compositional data into new coordinates, which follow the rules of Euclidean geometry in real space. The log-ratio transformations remove units of the parts involved, and then statistics and analysis results are done in the log-ratio coordinates which follow the rules of Euclidean geometry in real space (Blake et al., 2016). CoDa tools for the processing and transformation of compositional data are freely available through CoDaPack v.2.0. program (Comas-Cufí et al., 2011). In this study, the centered-log-ratio (clr) transformation was applied to the obtained CoDA matrix, *x*, of D parts as follows:

$$clr\left(x\right)={\left(ln\frac{x_i}{g\left(x\right)}\right)}_{i=1,\dots,D},where\;g\;\left(x\right)=\sqrt[D]{x1\ast x2\ast\dots\ast x_D}$$

In detail, the following statistical treatments were performed. Ionic balance errors for the data were calculated using PhreeqC (version 2.18) (Parkhurst and Appelo, 1999). Most samples had calculated ionic balance errors below the recommended standard of ± 5% (Freeze & Cherry, 1979). Only compositional hydrochemical and isotopic data were included for the statistical analysis (temperature, pH, and electrical conductivity were not included). In this way, a data matrix was created using major ions (Na^+^, Ca^2+^, Mg^2+^, K^+^, HCO_3_^−^, Cl − , SO_4_^2−^) containing 36 observations and 8 variables. In addition, to investigate statistically the isotopic nature of water samples, ternary principal component plots using isotopic abundance (δ^2^H, δ^18^O, and ^3^H) were prepared using CodaPack. The ternary principal component plots condense the compositional variability into a descriptive tool for identifying, in this case, the different water types on the relationships between the relative proportions of isotopic constituents. Any variables with a large proportion of samples below the LOQ (> 33% of samples below the LOQ) were discarded. A variation matrix (Aitchison, 1986) was generated to examine the data. Where each component of the variation matrix, *t*, describes the log relationship between two variables xi and xj.

This transformation was developed by Aitchison (1986) and is commonly used for covariance-based PCA (Drew et al., 2008; Engle & Blondes, 2014).

The MSA tools (e.g., principal component analysis) allow us to investigate the factors controlling the processes taking place in aquifers driving the hydrogeochemical composition of groundwater (Blake et al., 2016; Piña et al., 2018). The PCA is a statistical technique based on the estimation of new variables called principal components, and as linear combinations of the original variables that maximize the variance (Abdi & Williams, 2010). The definition of the number of retained components was made using the scree test (Cattell, 1966; Reimann et al., 2002), looking for the higher portion of the total variance represented on those components, and obtaining a more trustworthy representation of the variables involved (Blake et al., 2016) through the implementation of compositional covariance biplots. Their interpretation kept the rules adapted by Blake et al. (2016) as follows: (1) If two vertices are coincident or situated close to each other, they are proportional; (2) the length of a link between two vertices is proportional to the log ratio of those two variables; (3) if three or more vertices lie on the same link, they may represent a sub-composition with one single degree of freedom; and (4) if two links between four separate clr variables are orthogonal, then the corresponding pairs of variables may vary independently of each other (this also applies for two orthogonal links describing sub-compositions).

## Results

### Lithologic characterization

**Fig. 2:**
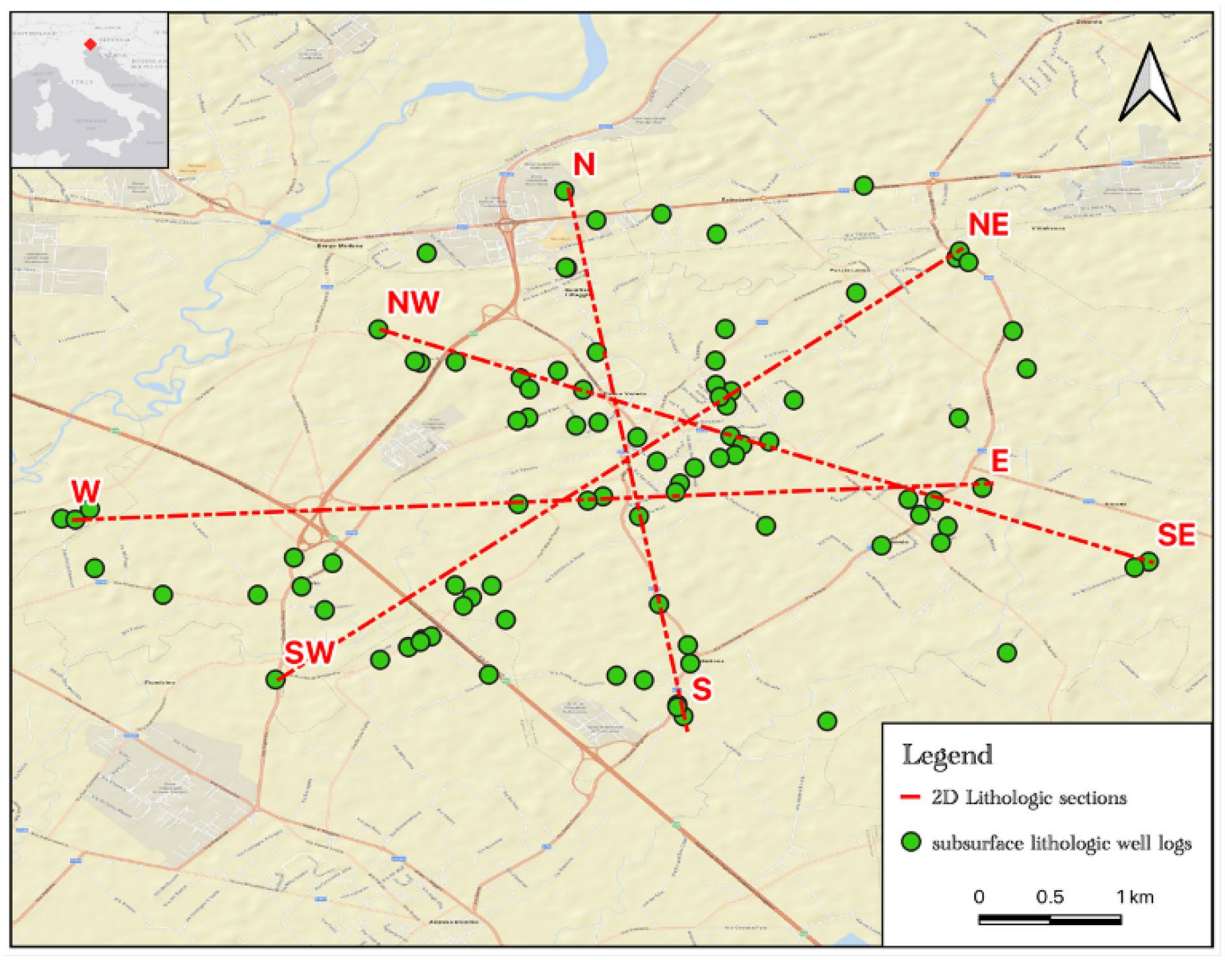
Location of the lithostratigraphic logs and the traces of the 2D sections

A detailed lithological characterization was carried out thanks to the many lithological data used in a limited area. In Fig. 2, it is possible to observe the location of the lithostratigraphic logs used for the realization of the lithological model and the traces of the 2D sections extrapolated from it. The model highlighted lithological characteristics typical of the low plain, that is thin layers of superimposed sandy/gravelly sediments immersed in an important clayey matrix. The subsoil of Fiume Veneto is very heterogeneous vertically with a certain horizontal continuity; only in some cases are there sandy/gravel layers with scarce lateral continuity.

**Fig. 3:**
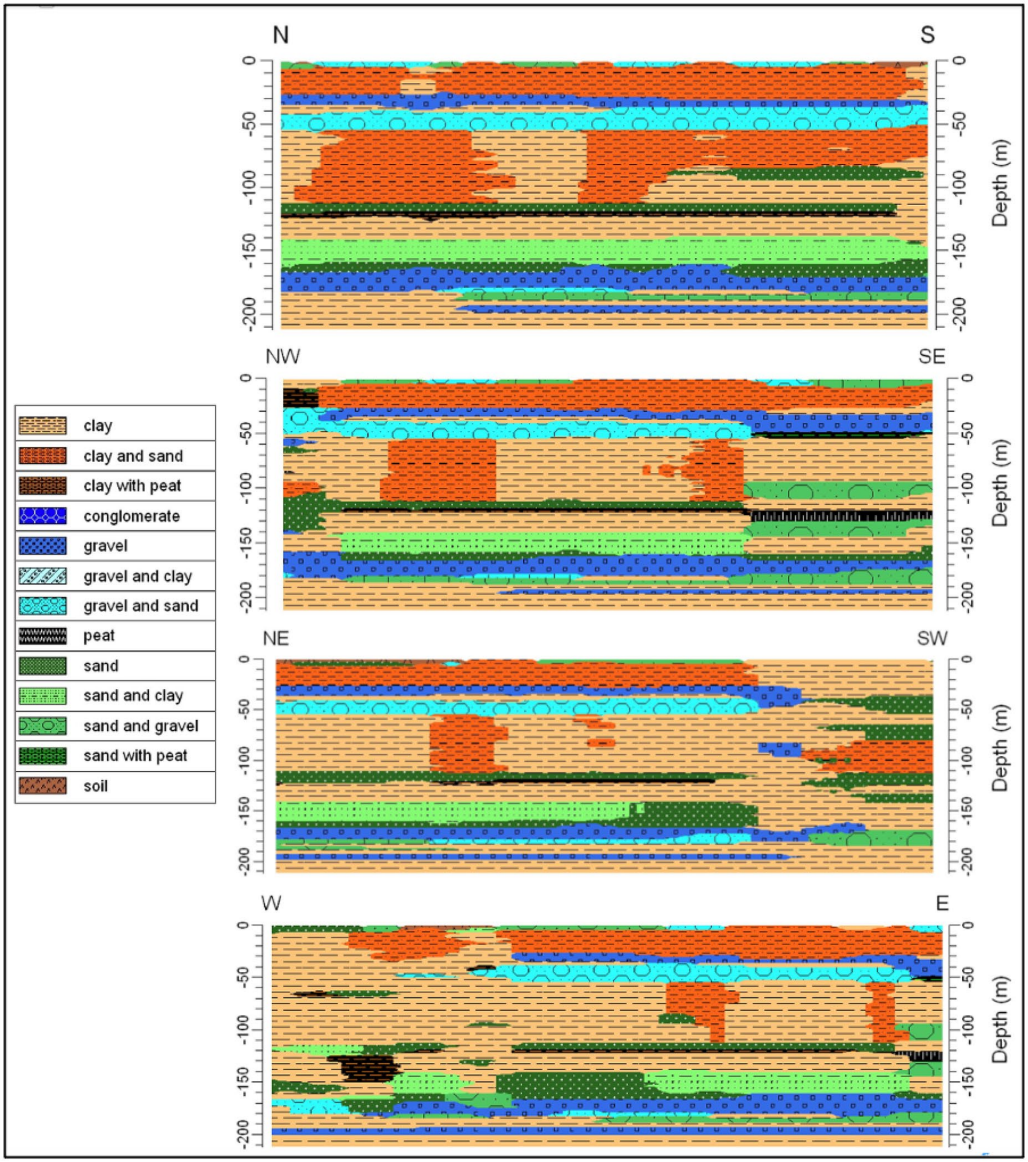
2D lithostratigraphic sections extrapolated from the lithological model

In accordance with the hydrogeological setting of the area, potential artesian aquifers are observed in Fig. 3. These aquifers present a marked lateral continuity, with the exception of a sandy layer located at a depth of about − 80/ − 100 m which is present only in the southern sector of the study area. In detail, the following potential aquifers have been identified:

- 0 m/ − 5 m, consisting mainly of soil, gravel, and sand;
 − 30 m/ − 50 m, consisting mainly of gravel and gravel with a sandy matrix;
 − 80 m/ − 90 m, consisting mainly of sand with an important horizontal heterogeneity. This layer is present only in the southern and southeastern area;
 − 110 m/ − 120 m, consisting mainly of sand;
 − 140 m/ − 180 m, the aquifer is divided into two sectors: (i) the upper part (up to − 160 m) mainly consisting of sand with a clayey matrix, (ii) the lower part consisting of gravel, sandy gravel;
 − 190 m/ − 200 m, consisting mainly of gravel.

### Hydrochemical characterization

The main chemical and physical parameters analyzed in all the water samples are shown in Table 1. The results allow differentiating the water samples into two main categories: superficial aquifer water (FVFR) from one side and all the other samples from the other side (FVAs, FVAd, FVF, and C).

The distribution of dissolved oxygen (DO) highlights that water samples directly in contact with the atmosphere show higher values with respect to superficial aquifer water samples which have less oxygenated waters given to the reduced contact with the atmosphere (Datry et al., 2004), and probably, from waters with greater age and stationing in the subsoil (Schmittner et al., 2007).

The water samples from the superficial aquifer (FVFR) show higher values of Na^+^ (between 3.3 and 8 mg/L), K^+^ (between 2 and 4.7 mg/L with a peak at 10.2 mg/L in the FVFR4 sample), and Ca^2+^ (between 68 and 132.8 mg/L). Regarding Mg^2+^ values, it varies between 12 and 38 mg/L, lowers with respect to intermediate and deep aquifer water samples (FVAs and FVAd), but are similar to wastewater (FVF) and canal (C) samples. In detail, wastewater samples show values < 15 mg/L, while canal samples have slightly higher values (between 16.6 and 17.2 mg/L). Cl^−^ shows variation between 2.6 and 6.9 mg/L in superficial aquifer water samples (FVFR), which is higher with respect to intermediate and deep aquifer water samples, but lower values of sulfates. SO_4_^2−^ concentrations show average values of 19.9 mg/L for superficial aquifer water samples (FVFR) and higher values for all the other water types, in particular 67.5 mg/L, 72.5 mg/L, and 86.1 mg/L for intermediate and deep aquifer water (FVAs and FVAd), wastewater (FVF), and canal (C) respectively. Finally, the values of the bicarbonate ion are higher in superficial aquifer water samples (FVFR) with respect to the intermediate and deep aquifer water samples (around 420 mg/L) (Balestra et al., 2022).

On the opposite, both the intermediate and deep aquifer water samples (FVA) show different trends of the same element analyzed: lower values of Na^+^ (< 5 mg/L), K^+^ (< 1 mg/L), and Ca^2+^ (29 and 66.9 mg/L). In addition, these samples have higher Mg^2+^ values than all the others. As regards anionic elements, Cl^−^ has very low values in both the intermediate and deep aquifer water samples (FVA) for which the maximum concentration is around 2.2 mg/L, and high values of SO_4_^2−^, which could be due to the dissolution of deep aquifer lithologies characterized by important concentrations of soluble minerals (Sharma & Kumar, 2020). Finally, the values of the bicarbonate ion are less than 180 mg/L, which is for both the intermediate and deep aquifer water samples. The superficial aquifer water reaches 420 mg/L (Binda et al., 2022).

**Fig. 4:**
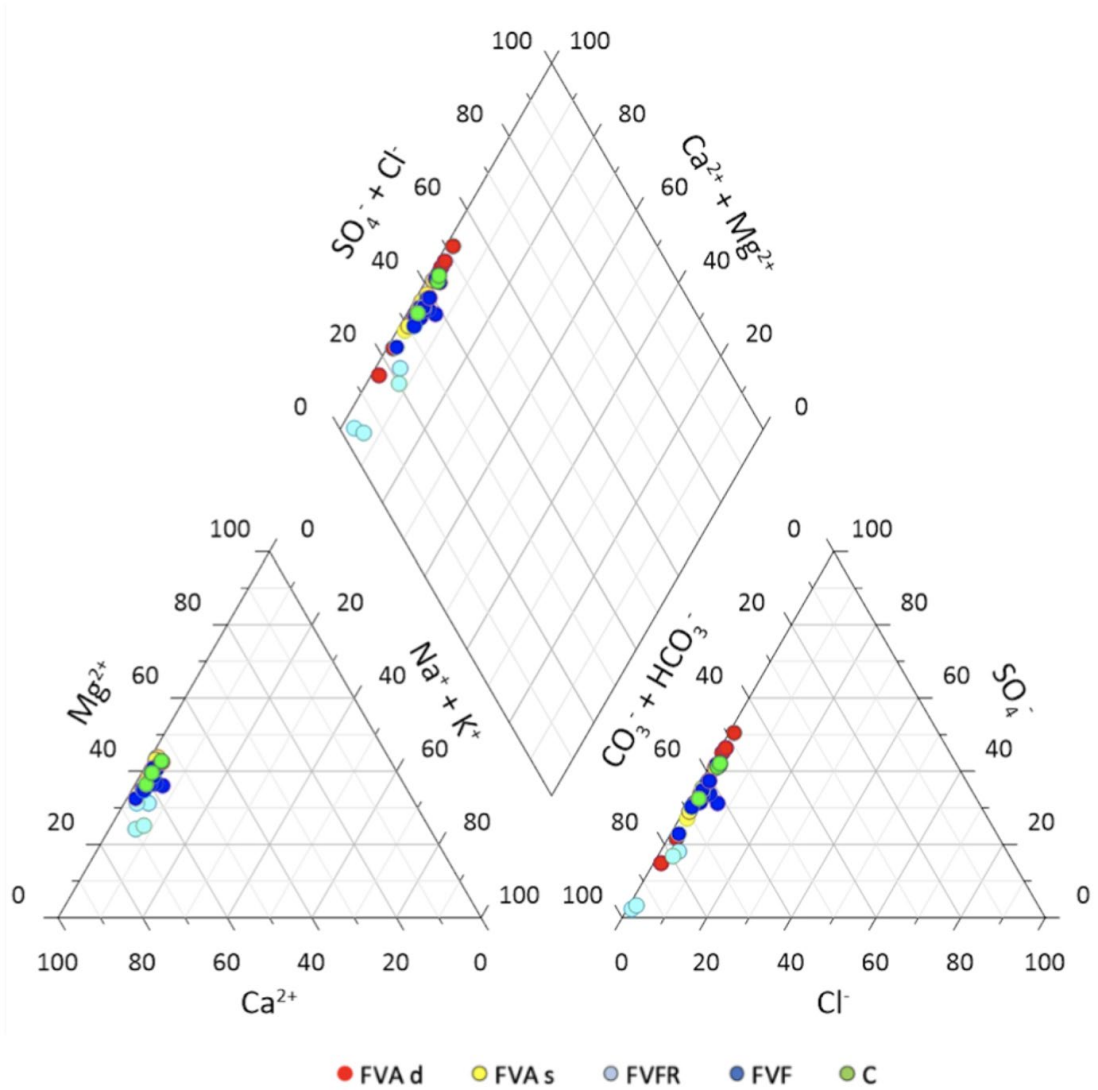
Piper diagram of the data collected in the sampling site, using Grapher 10 software. Colored in light blue superficial aquifer water samples (FVFR), in yellow intermediate aquifer water samples (FVAs), in red deep aquifer water samples (FVAd), in blue wastewater samples (FVF), in green the surface canal water samples (C)

All the data obtained were used in the Piper diagram to identify the origin of the water samples based on the results obtained by chemical analysis (Fig. 4). Without it, the superficial aquifer water samples (FVFR, colored in light blue) have dominant bicarbonate-alkaline earthy facies: bicarbonate-calcium with a prevalence of the Ca^2+^ ion from the Mg^2+^ one. These waters are probably mainly generated by chemical processes linked to the dissolution of carbonates by rainwater (Madonia et al., 2020). Nevertheless, another cluster can be observed which groups the other types of waters (both the intermediate and the deep aquifer water samples, wastewater, and water from surface canals). These water samples show a general increase in SO_4_^2−^ and a less marked increase in Mg^2+^. Given the marked increase in SO_4_^2−^, these waters can be traced back to the mixing of bicarbonate-calcium and sulfate–calcium waters.

From the qualitative analysis, it is possible to identify two clusters of water, one mainly bicarbonate-calcium and the other which has an increase in SO_4_^2−^ (mix with sulfate–calcium waters).

In the ternary diagrams at the bottom of the graph in Fig. 4, the increase in HCO_3_^−^ and alkali for superficial aquifer water samples (FVFR) and SO_4_^2−^ and Mg^2+^ for FVA, FVF, and C water samples can be observed in more detail, highlighting the difference between the two clusters: superficial aquifer water (FVFR) form one side and all the other samples from the other side (FVAs, FVAd, FVF, and C).

**Fig. 5:**
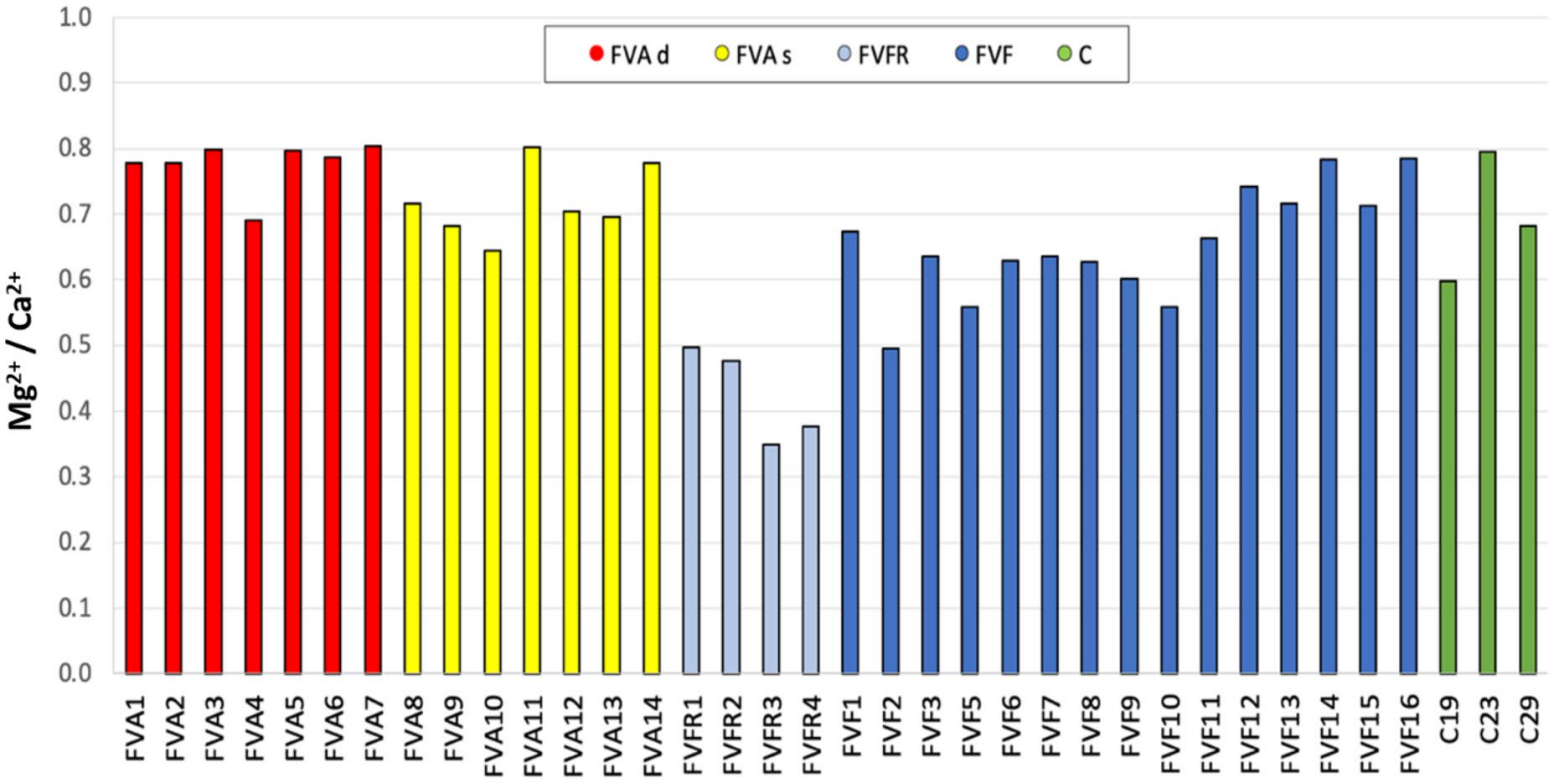
Ratio Mg^2+^/Ca.^2+^ obtained in all the samples analyzed. Colored in light blue superficial aquifer water samples (FVFR), in yellow intermediate aquifer water samples (FVAs), in red deep aquifer water samples (FVAd), in blue wastewater samples (FVF), in green the surface canal water samples (C)

In addition to the evaluation of the chemistry of water samples, it is possible to study and interpret the water–rock interaction through the relationships between some elements significantly present in the water (Jia et al., 2020; Kelemen et al., 2019). In this case study, the most important ratio is the Mg^2+^/Ca^2+^. The literature shows that minimum values of the Mg^2+^/Ca^2+^ ratio occur in surface runoff waters and in fast-flowing groundwaters such as in karst ducts (Yalkowsky et al., 2010). Figure 5 shows the Mg^2+^/Ca^2+^ ratio of the sampled waters, which is higher than 0.5 for all the samples except for superficial aquifer water samples (FVFR).

It can be hypothesized that the high Mg^2+^/Ca^2+^ ratio of the FVA, FVF, and C water samples may be due to the lithological nature of the Fiume Veneto area. Furthermore, regarding both the intermediate and the deep aquifer water samples (FVA), the Mg^2+^/Ca^2+^ ratio tends to increase with depth, from intermediate aquifer waters (at about 30–80 m, FVAs) to deep aquifer waters (at about 180 m, FVAd), probably due to a decrease in Ca^2+^ concentrations with increasing depth probably due to differences in the chemistry of sediments (de Ridder et al., 2018). This can be explained by the lower availability of CO_2_ at depth as well as from ionic exchanges between the Ca^2+^ and Na^+^ and K^+^ (Cucchi et al., 2008; Zini et al., 2009).

Anionic and cationic analysis shows two different clusters: superficial aquifer water (FVFR) from one side and all the other samples from the other side (FVAs, FVAd, FVF, and C).

**Fig. 6:**
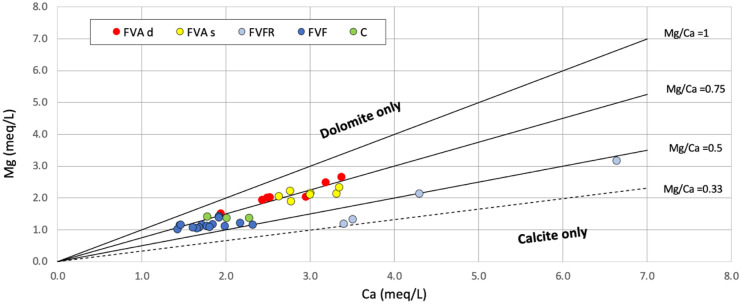
Water sample differentiation is based on the value of the Mg^2+^/Ca^2+^ ratio (Mg^2+^ and Ca^2+^ expressed in meq/L). The Mg^2+^/Ca^2+^ ratio higher than 1 indicates dolomite affinity; Mg^2+^/Ca.^2+^ less than 0.33 indicates calcite affinity. Colored in light blue superficial aquifer water samples (FVFR), in yellow intermediate aquifer water samples (FVAs), in red deep aquifer water samples (FVAd), in blue wastewater samples (FVF), in green the surface canal water samples (C)

Figure 6 shows the distribution of the water samples based on the content of Mg^2+^ and Ca^2+^ and their ratio. The weathering of dolomite in the carbonate system contributes the majority of Mg^2+^, in which the Mg^2+^/Ca^2+^ molar ratio indicates the relative proportions of calcite and/or dolomite dissolution (Zavadlav et al., 2013). Dissolution of calcite produces waters with a Mg^2+^/Ca^2+^ molar ratio of less than 0.1, equal to 0.33 in the case of congruent dissolution of calcite and dolomite, and equal to 1 if only dolomite is dissolving (Szramek et al., 2011). The graph shows that the superficial aquifer water samples (FVFR, colored in light blue) have a Ca^2+^ affinity with an Mg^2+^/Ca^2+^ molar ratio in meq/L < 0.5, while the samples of both the intermediate and the deep aquifer water (FVA), wastewater (FVF), and surface canals (C) have a dolomitic footprint (Mg^2+^/Ca^2+^ meq/L between 0.5 and 1).

Also in this case, the Mg^2+^/Ca^2+^ molar ratio indicates two different clusters: superficial aquifer water (FVFR) from one side and all the other samples from the other side (FVAs, FVAd, FVF, and C).

### Stable isotopes analysis

**Fig. 7:**
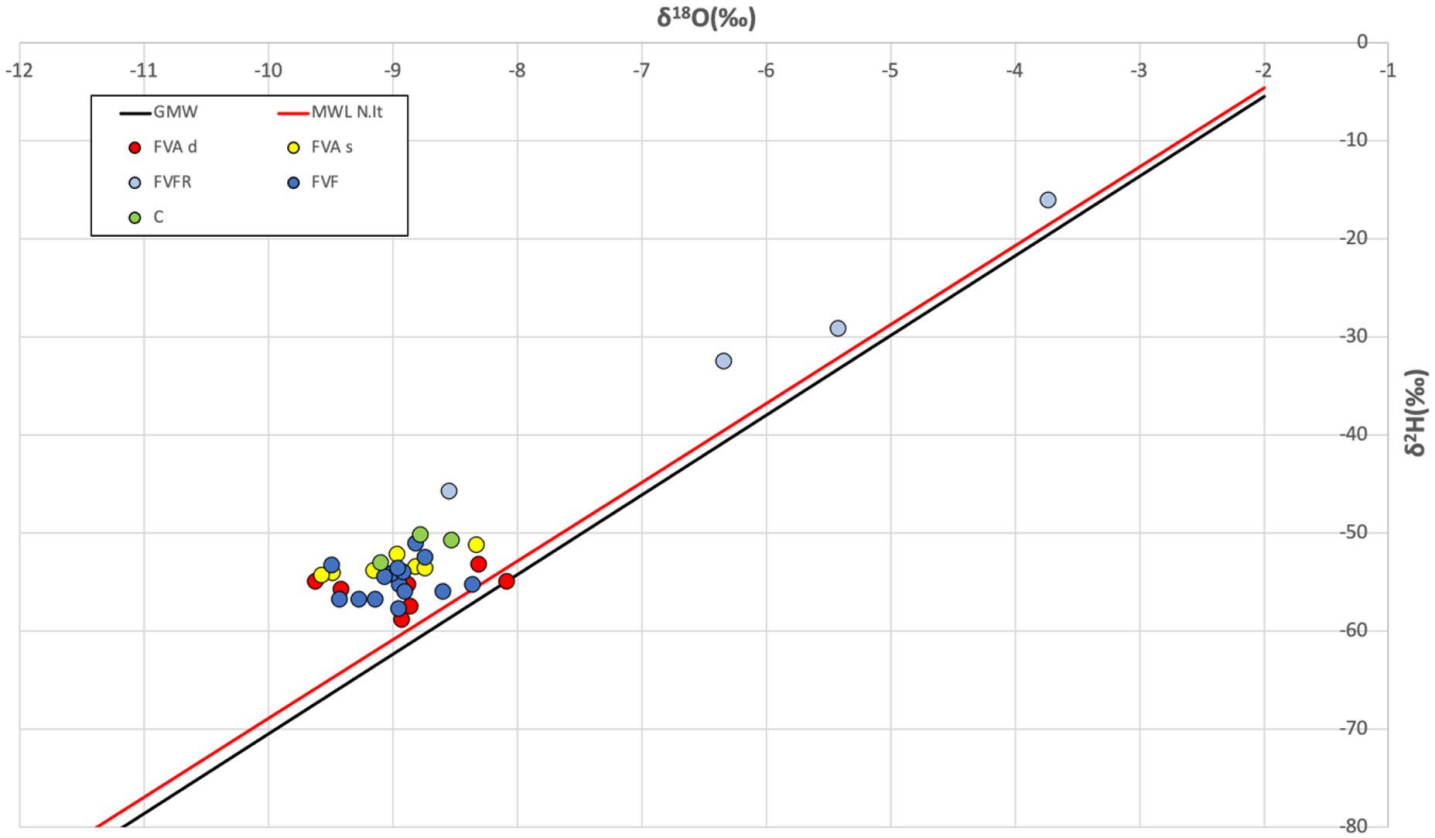
Isotopic composition of δ^2^H and.^18^O on the analyzed samples. Meteoric water lines are also reported for comparison: the black line represents the Global Meteoric Water Line (GMWL; Craig, 1961); the red line represents the Local Meteoric Water Line (LMWL), defined for Northern Italy (Longinelli & Selmo, 2003). Colored in light blue superficial aquifer water samples (FVFR), in yellow intermediate aquifer water samples (FVAs), in red deep aquifer water samples (FVAd), in blue wastewater samples (FVF), in green the surface canal water samples (C)

Stable isotopes are important tracers in water and indicators of climatic variability (Roberts et al., 2010; Yang et al., 2019). Figure 7 shows how superficial aquifer water samples (FVFR) follow the trend of the meteoric lines, which have a direct recharge and, consequently, are less depleted in δ^18^O than δ^2^H compared to both the intermediate and the deep aquifer waters (FVA). All the other types of water (FVA, FVF, and C) are in a separate group characterized by values between − 50 and − 60‰ for δ^2^H and values between − 8 and − 10‰ for δ^18^O.

In this isotopic study, it is shown how the wastewater (FVF), as well as the waters of surface canals (C), is mainly grouped with the intermediate and deep aquifer waters (FVAd and FVAs), while the superficial aquifer water samples (FVFR) follow a different trend, highlighting the two main different clusters of superficial aquifer water (FVFR) from one side and all the other samples from the other side (FVAs, FVAd, FVF, and C).

### Tritium analysis

The statistics (minimum and maximum value, mean, median, standard deviation, interquartile range, upper quartile, and lower quartile) of the ^3^H data obtained are reported in Table 2. The statistical analysis was performed with the aim of having a general description of the entire data set and to have initial feedback on the isotopic characteristics of the different types of water samples collected.

**Table 2:** Statistics on ^3^H concentration analyzed in the different types of water

	Mean	Median	Min	Max	Lower quartile	Upper quartile	IQR	Std dev	Variance
FVFR	6.27	6.33	5.17	7.23	5.72	6.88	1.16	0.91	0.83
FVAs	3.54	3.65	2.66	4.21	3.14	4.01	0.87	0.60	0.36
FVAd	3.26	3.31	2.07	4.24	2.71	3.91	1.21	0.82	0.68
FVF	3.82	3.81	3.11	4.97	3.50	4.05	0.55	0.46	0.21
C	4.56	5.05	3.42	5.21	4.23	5.13	0.89	0.99	0.98

FVFR means superficial aquifer water samples, FVAs means intermediate aquifer water samples, FVAd means deep aquifer water samples, FVF means wastewater samples, and C means the surface canal water samples

The samples are classified into four categories based on the average residence times of water samples, referring to the classification of Clark and Fritz (1997):

-  < 0.8 TU: water samples with ^3^H detected with low values above the detection threshold. These samples characterize long-recharge aquifers with estimated ages between 50 and 70 years;
- 0.8 TU < ^3^H < 5 TU: water samples with an estimated age between 10 and 50 years (mixing of modern and old/sub-modern waters);
- 5 TU < ^3^H < 15 TU: water samples with an estimated age between 1 and 10 years (recent recharge);
-  > 15 TU: water samples affected by the possible presence of water contamination phenomena (external sources of.^3^H).

**Fig. 8:**
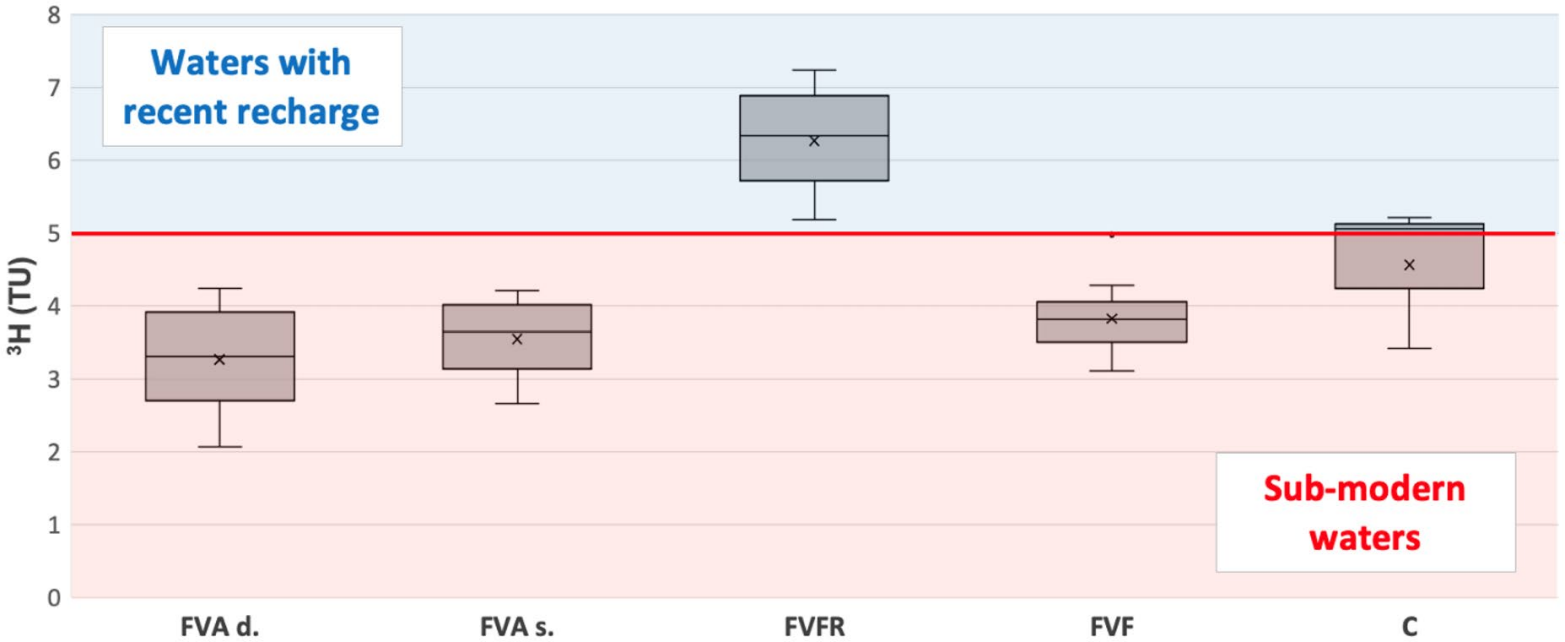
Box plot on ^3^H concentration in all the samples analyzed. FVFR means superficial aquifer water samples, FVAs means intermediate aquifer water samples, FVAd means deep aquifer water samples, FVF means wastewater samples, and C means the surface canal water samples

The average ^3^H value of all the samples analyzed is between 3.82 and 6.27 TU, as shown in the box plot of Fig. 8, in which two different clusters are observed: water with recent recharge (colored in light blue in Fig. 8) and sub-modern water (colored in red in Fig. 8).

Both the intermediate and the deep aquifer waters (FVAs and FVAd) and wastewaters (FVF) are identified as sub-modern waters with low values of ^3^H ranging between 3 and 4.5 TU, which is lower than the isotopic footprint of the meteoric precipitation, suggesting a longer residence time in the subsoil with an estimated age between 10 and 50 years. Generally, in complex multi-aquifer systems, the progressive decrease of ^3^H in the waters from the recharge areas to the depth is linked to the nature of the aquifers with regional water circulation (Cao et al., 2020; Wilske et al., 2020).

On the opposite, superficial aquifer water samples (FVFR) are classified as modern water with recent recharge and with a ^3^H value between 5 and 7 TU.

Finally, the waters of the surface canals (C) show values attributable to sub-modern waters but with slightly higher values of ^3^H (between 4 and 5 TU), probably relating to a possible mixing with modern waters due to their presence on the surface and direct contact with the atmosphere, as described in Mahlangu et al. (2020) and Malov (2021).

According to this result, the ^3^H values identify two main clusters: superficial aquifer water (FVFR) from one side and all the other samples from the other side (FVAs, FVAd, FVF, and C), confirming the previous analyses.

Furthermore, the ^3^H values in the samples collected in the Fiume Veneto area in the intermediate aquifer, deep aquifer, wastewater, and canal waters reflect the values determined by Mayer et al. (2014) in the adjacent Veneto plain, between the Brenta and Piave rivers, where the aquifers at medium depth (about 150–180 m a.s.l.) have been defined as regional aquifers not directly influenced by rainwater and older waters belonging to deeper aquifers.

The results obtained are also highlighted by comparing the ^3^H value with the average annual concentrations in rainwater recorded at the Locarno station in the north part of the Lago Maggiore in the south part of Swiss on the border with Italy (Global Network for Isotopes in Precipitation, “GNIP” station network). The average value of ^3^H, expressed in TU, in the precipitations into the period 1998–2008 varies from a minimum of 5.43 TU to a maximum of about 11.21 TU, with an annual average of 5.43 TU (IAEA/WMO, 2022). Therefore, making a comparison with the ^3^H value obtained in the analyzed samples, it is possible to distinguish the sub-modern waters from the waters with recent recharge (Table 3), further confirming the data obtained. The superficial aquifer water samples (FVFR), with an average value of 6.27 TU, are the only ones that present a meteoric footprint, further confirmed by the comparison with the ^3^H data, equal to 5.43 TU, recorded by IAEA/WMO (2022) at Locarno station from 1998 to 2008.

**Table 3:** Comparison between the average concentrations of ^3^H, expressed in TU, detected in the water samples analyzed and the average concentration of ^3^H in the precipitation recorded at the Locarno station (GNIP station network) in the period 1998–2008 (IAEA/WMO, 2022)

	Min	Mean	Max	Std dev
FVFR	5.17	6.27	7.23	0.83
FVAs	2.66	3.54	4.21	0.36
FVAd	2.07	3.26	4.24	0.68
FVF	3.11	3.82	4.97	0.21
C	3.42	4.56	5.21	0.98
GNIP Locarno station	5.43	7.90	11.21	1.72

*Min*, minimum value; *Max*, maximum value; *Std dev*, standard deviation. FVFR means superficial aquifer water samples, FVAs means intermediate aquifer water samples, FVAd means deep aquifer water samples, FVF means wastewater samples, and C means the surface canal water samples

**Fig. 9:**
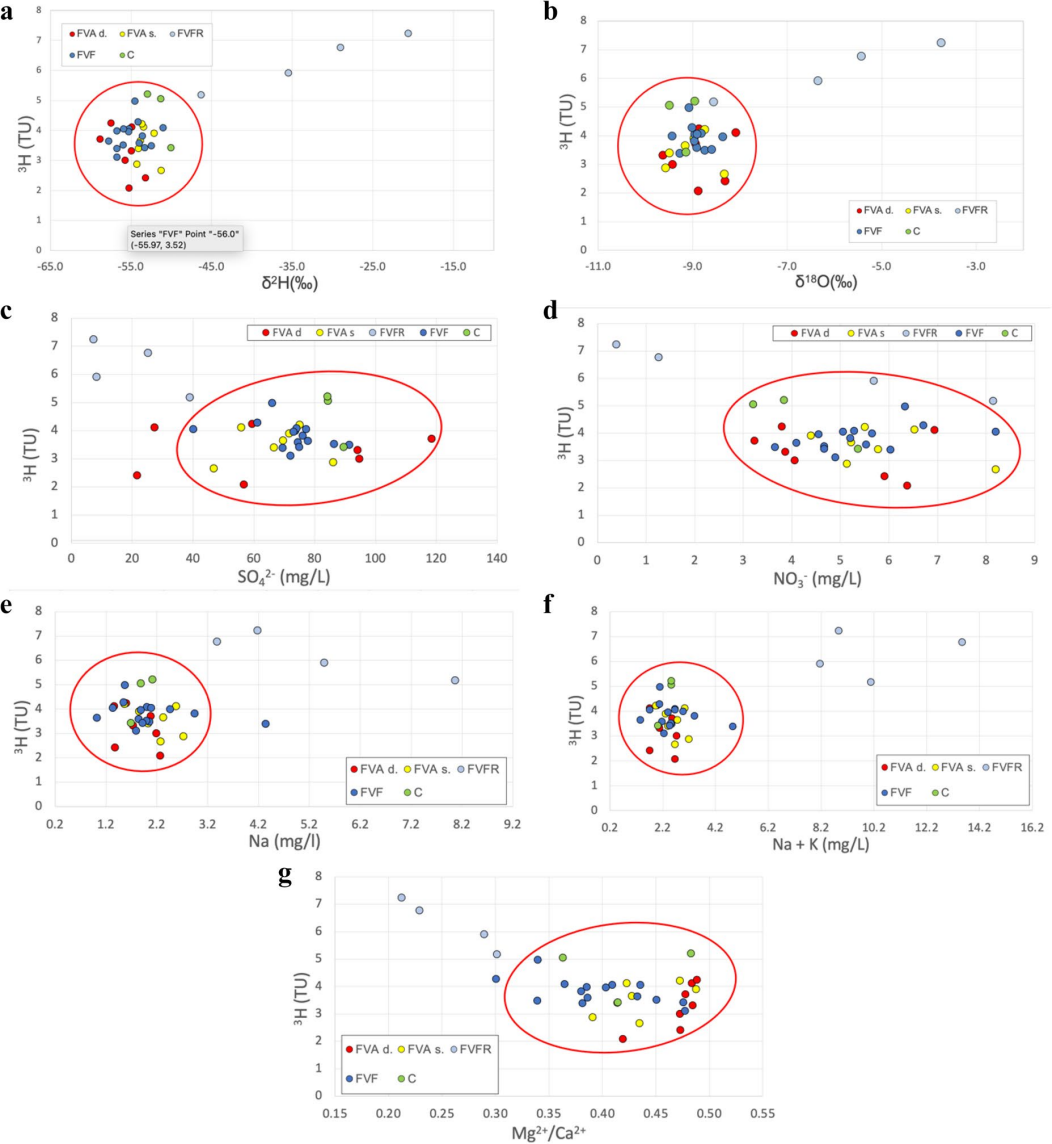
Correlation diagram: **a** ^3^H and δ^2^H; **b** ^3^H and δ^18^O; **c** ^3^H and SO_4_^2−^; **d** ^3^H and NO_3_^−^; **e** ^3^H and Na; **f** ^3^H and Na + K; **g** ^3^H and Mg^2+^/Ca.^2+^. Colored in light blue superficial aquifer water samples (FVFR), in yellow intermediate aquifer water samples (FVAs), in red deep aquifer water samples (FVAd), in blue wastewater samples (FVF), in green the surface canal water samples (C)

Figure 9 shows the superficial aquifer water samples (FVFR), classified by the ^3^H values as modern waters, enriched in δ^2^H (Fig. 9a) and δ^18^O (Fig. 9b) reflecting their predominantly meteoric nature (as previously shown in Fig. 7, in which these samples are located along the line of local rainwater). While the remaining types of water (both the intermediate and the deep aquifer water, wastewater, and canal water samples), classified by the ^3^H values as sub-modern waters, are grouped in a single cluster indicating a similar nature (inside the red circle in both Fig. 9 a and b).

The result obtained by correlating ^3^H with δ^18^O is important. In fact, it can be seen in Fig. 9b how the wastewater (FVF) and the waters of the superficial canals (C) have the same isotopic footprint as the intermediate and deep aquifer waters (FVAd and FVAs). The superficial aquifer water samples (FVFR) are also in another separate cluster characterized by high values of δ^18^O.

In addition, due to the important presence of SO_4_^2−^ in the superficial aquifer water samples (FVFR) analyzed in the Fiume Veneto area, a comparison between the value of ^3^H and the concentration of SO_4_^2−^ is shown in Fig. 9c, with the aim of identifying further differences between the different types of water collected. Figure 9c highlights the lower value of SO_4_^2−^ and high values of ^3^H in the superficial aquifer water samples (FVFR), while the remaining samples have values of SO_4_^2−^ between 60 and 100 mg/L and values of ^3^H generally < 4 TU. This suggests that wastewater (FVF) and surface canals water (C) are very similar from a geochemical and isotopic point of view to the intermediate and the deep aquifer water samples (FVAs and FVAd respectively), which are rich in SO_4_^2−^ probably due to the dissolution of aquifers, while superficial aquifer water samples (FVFR) show marked discrepancies.

**Fig. 10:**
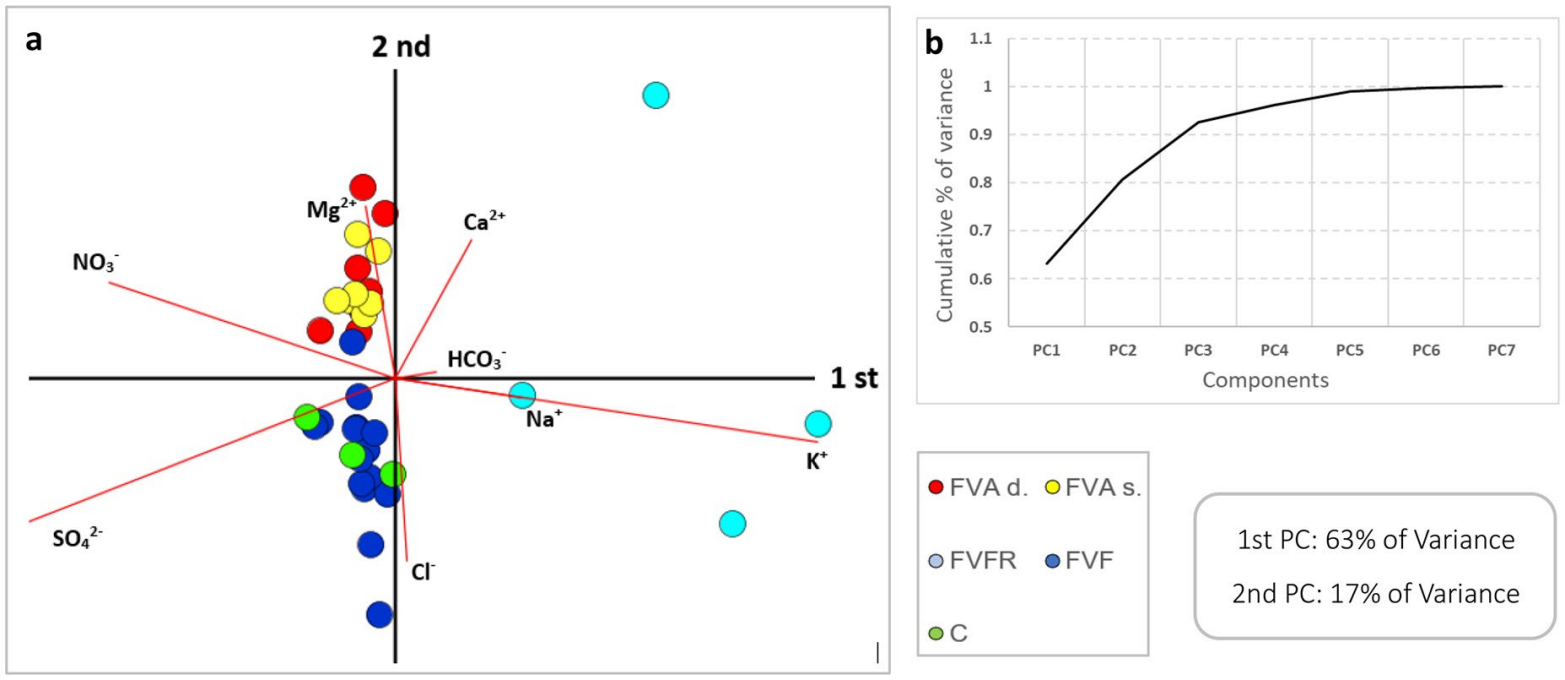
Compositional PCA biplots for compositional hydrochemical data. **a** a centered-log ratio (clr) biplot of the data set; **b** scree plots of the variance represented by each principal component for the data matrix. Elaborations were conducted using the CodaPack program. Colored in light blue superficial aquifer water samples (FVFR), in yellow intermediate aquifer water samples (FVAs), in red deep aquifer water samples (FVAd), in blue wastewater samples (FVF), in green the surface canal water samples (C)

The correlation diagram of ^3^H and NO_3_^−^ (Fig. 10d) is similar to that of ^3^H and SO_4_^2−^ (Fig. 9c), but with superficial aquifer water samples (FVFR) characterized by lower values of nitrate with respect to the other water samples.

The correlation diagrams of ^3^H and Na (Fig. 9e) and ^3^H and Na + K (Fig. 9f) are very similar to each other, but in Fig. 9f, the superficial aquifer water samples differ even more from the other water samples.

Finally, the comparison between ^3^H and Mg^2+^/Ca^2+^ (Fig. 9f) gives a correlation diagram similar to that of ^3^H and SO_4_^2−^ (Fig. 9c). The wastewater samples (FVF) differ well from the intermediate and deep aquifer water ones, especially from the deep aquifer water samples (FVAd).

All the correlation diagrams in Fig. 9 highlight the presence of two main clusters: one characterized by superficial aquifer water samples (FVFR) and the second by all the other types of water samples collected (FVAs, FVAd, FVF, and C).

### Statistical analysis

The principal component analyses (PCA) allowed us to identify the hydrogeochemical processes that control the main concentrations of ions in different types of water. The results of the PCA were conducted using the CodaPack program. The clr biplots obtained from the PCA are presented in Fig. 10. The scree plot of variance in Fig. 10b “breaks” after the second component indicating that a biplot of the first two PCs will provide a trustworthy representation of the data (they represent 80% of the total log-ratio (clr) variance in the dataset). Generally, the compositional covariance biplot (Fig. 10a) shows the highest clr variances for K, SO_4_^2−^ and NO_3_^−^, followed by Cl, Ca^2+^, and Mg^2+^, and the lowest clr variances for Na^+^, HCO_3_^−^.

The PCA results show that constituents with positive PC1 values are dominant for FVFR waters. They are located in the eastern quadrants of the biplot and fall within sub-composition [K^+^, Na^+^, HCO_3_^−^, Ca^2+^]. The remaining water samples, in contrast, show negative PC1 values. They are located in the western quadrants of the biplot and fall within two sub-compositions, [SO_4_^2−^, NO_3_^−^, Mg^2+^, Ca^2+^] and [SO_4_^2−^, Cl^−^]. This subdivision for FVAd-FVAs and FVF-C is well visible with PC2 values. The results of two PCAs allowed us to identify processes that are likely to control the hydrochemistry of the water samples examined.

The PCA obtained, whose results support the hypothesis of two processes, mainly relating to phenomena of direct mixing with rainfall (described by PC1 with 48% of the observed variance) that could be linked to high values of 3H and for superficial aquifer water samples (FVFR), and the different nature of the water samples (PC2, described with 17% of the observed variance) which could be connected to different evolutionary processes. However, this process is influenced by the mixing of waters of different natures in some samples.

The PC1 supports the hypothesis of a different nature of water–rock interaction that influences the hydrogeochemical evolution of the water samples (described with 63% of the observed variance). The FVFR samples that circulate in the shallow aquifer (constituted of sandy silts and silty sand deposits) are enriched in alkalis. The FVAd, FVAs, FVF, and C, in contrast, present an enrichment in sulfate-carbonate ions due to different aquifer lithologic compositions, mainly dolomite-carbonate composition. PC2, on the other hand, support anthropic influence on waters, mainly for FVF and C samples (described with 17% of the observed variance). Although it has been seen that the geochemical-isotopic nature of the FVA, FVF, and C samples was very similar, the Cl^−^ ion enrichment for FVF and C samples may be due to mild mixing with chlorinated water deriving from the discharge of the houses present in the study area.

**Fig. 11:**
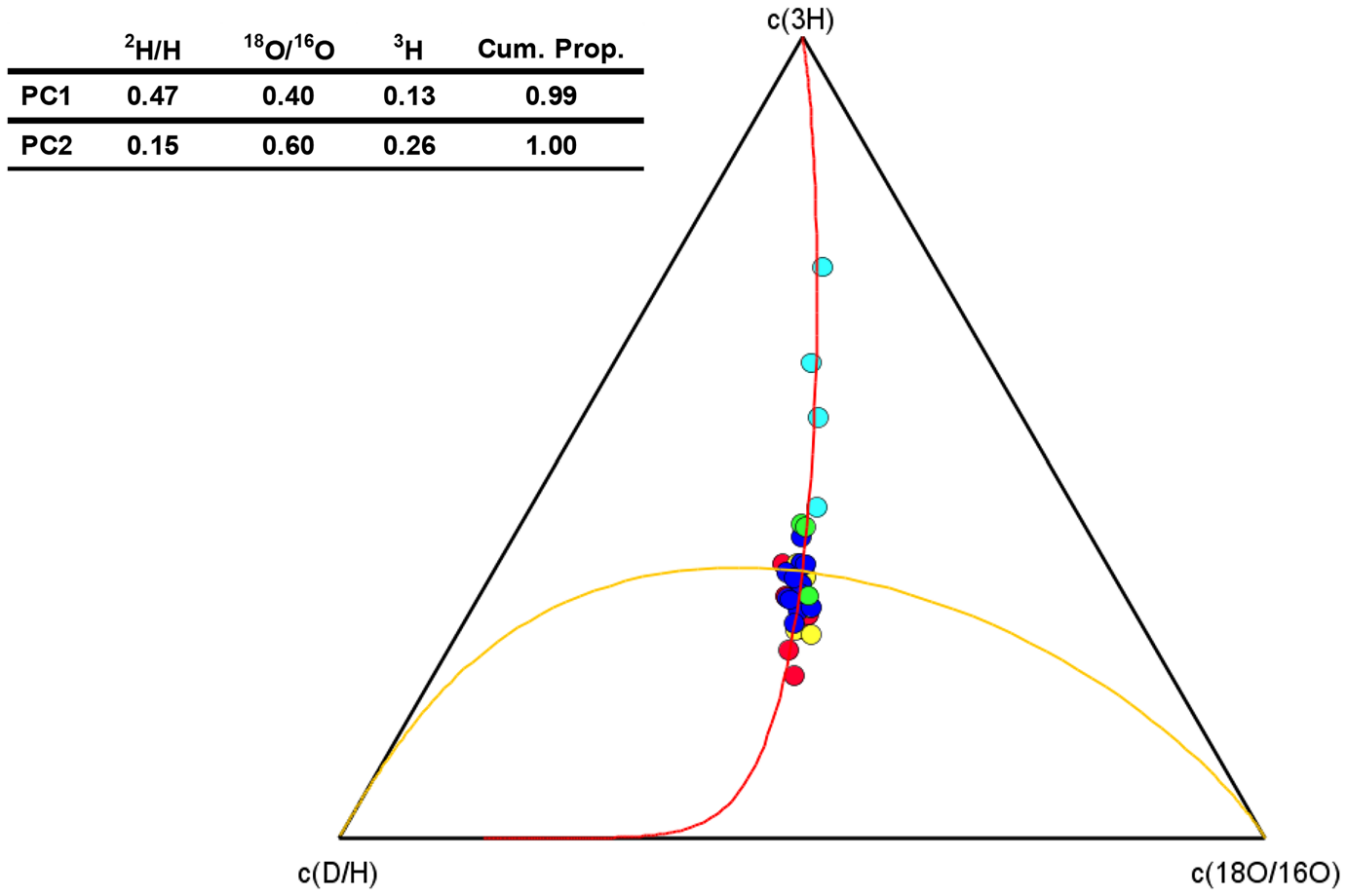
Ternary principal components plot ^2^H, ^18^O, and.^3^H. Colored in light blue superficial aquifer water samples (FVFR), in yellow intermediate aquifer water samples (FVAs), in red deep aquifer water samples (FVAd), in blue wastewater samples (FVF), in green the surface canal water samples (C)

The ternary principal component plots of isotopic data show as all samples were much more affected by PC1 than by PC2. More specifically, 99% of the samples seemed to be influenced dominantly by the same isotopic footprint. However, while all samples have the same isotope signature of ^2^H and ^18^O, the tritium concentrations differentiate the FVFR samples very well from the rest of the samples (Fig. 11). This result further confirms a meteoric origin of the FVFR samples.

## Discussion

Geochemical and isotopic analysis was used to investigate the origin of the water samples collected and analyzed, discriminating the different types of water, and understanding their interactions. This approach is widely used in literature especially to date water resources with wide applications both in Italy (Mayer et al., 2014; Romano et al., 2018; Telloli et al., 2022), in Europe (Balocchi et al., 2022; Brkić et al., 2020; Malov, 2021), and all over the word (Copia et al., 2020; Varol et al., 2020).

The results of stable and radioactive isotope analyses distinguish the presence of two main clusters of water aquifer, which are the most important water resources in the Fiume Veneto area, and which need to be protected due to climate change and to the water needs.

In the sampling site investigated, there are a lot of domestic water wells that have not been reported to the competent authorities. The domestic well data present in the public geodatabase are a close underestimation of the real situation representing the best knowledge framework (Zini et al., 2013). The number of wells reported to authorities is still lower than the actual one, as the transposition of Article 10 of the Legislative Decree 275 (1993) by the population remained largely disregarded (Zini et al., 2011). This is important to know in order to better understand critical aquifer depletion values.

The water-budget scheme of the Friuli-Venezia Giulia region proposed by Zini et al. (2011) shows a slightly positive water budget (+ 2.6 m^3^/s) which could be lower, or even negative, if we consider the withdrawals from wells not reported. However, the water budget is not homogeneous in the Friuli-Venezia Giulia region. In detail, in the Friuli plain on the right hydrographic of the Tagliamento river (Fiume Veneto area), Zini et al. (2011) have estimated that the discharges of the springs belt are a total of 45.9 m^3^/s of the 58.4 m^3^/s deriving from the high plain. From the difference between the inputs of the high plain and the springs belt discharges, was estimated the input of the low plain as 12.5 m^3^/s. Considering a withdrawal from aquifers equal to about 31.7 m^3^/s, the water budget in this area of the plain (and also in the Fiume Veneto area) is greatly affected by the overexploitation of the aquifers.

In addition, considering the data surveyed in the last decades by Granati et al. (2000), a mean discharge per artesian well of 0.8 l/s has been estimated.

According to this research, the total withdrawal in the Friuli-Venezia Giulia Region is about 62.4 m^3^/s. The total withdrawal amount from the confined aquifer systems is 44.4 m^3^/s, of which 3.3 m^3^/s in the high plain and 41.1 m^3^/s in the low plain areas (38.6 m^3^/s in Friuli-Venezia Giulia Region and 2.5 m^3^/s in Veneto Region, which is closed to Friuli-Venezia Giulia Region). So, almost 50% of the withdrawals are in the aquifer systems (Zini et al., 2013).

**Fig. 12:**
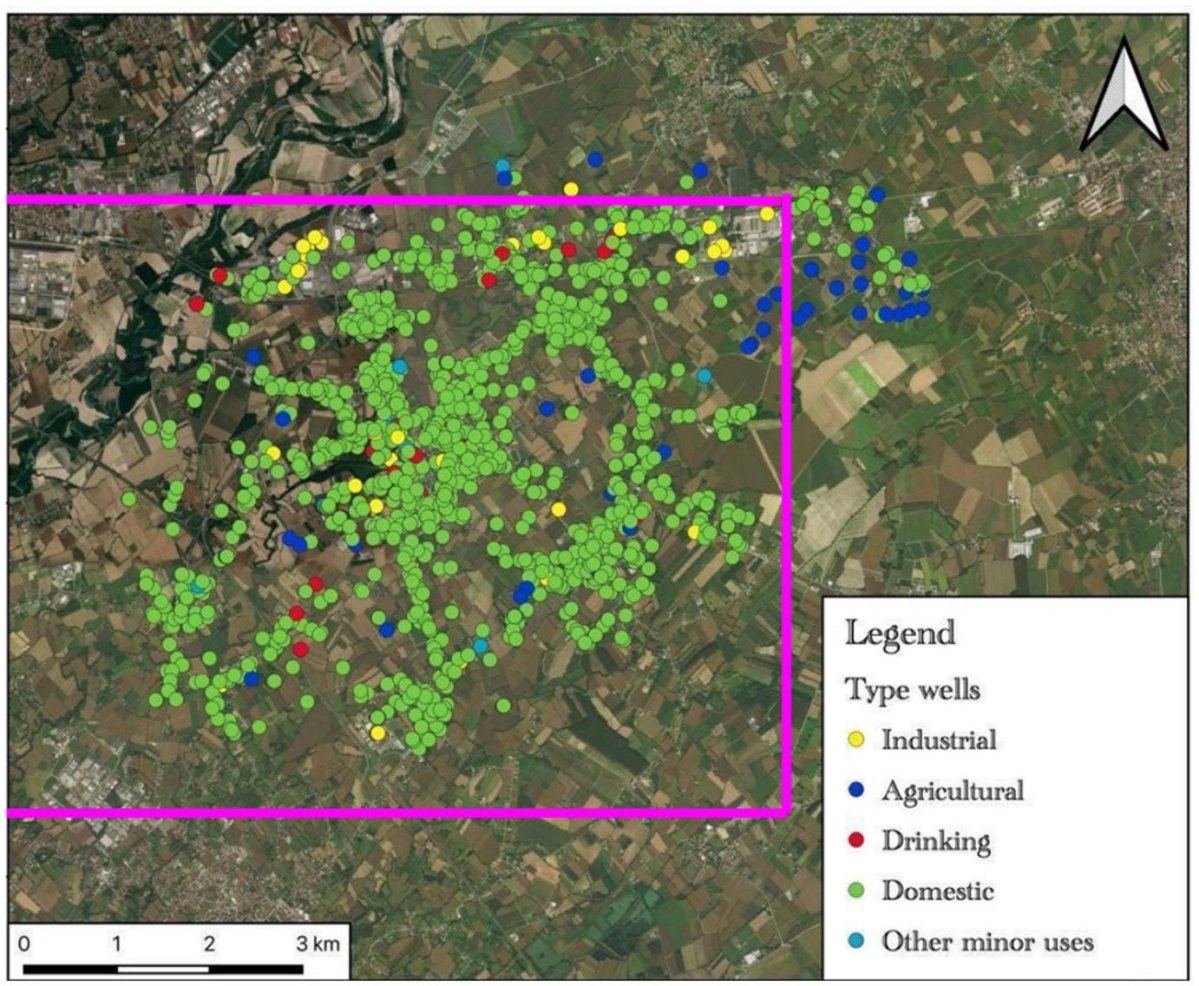
Map of the water wells reported to the authorities. Colored in yellow are the industrial wells; in blue are wells for agricultural practices; in red are drinking wells; in green are the domestic and private wells and finally in light blue are other minor uses. The pink lines delimitate the Fiume Veneto sampling area of the research study

Figure 12 shows the map of the water wells reported to the authorities. The Fiume Veneto area has a lot of domestic wells (1861—colored in green in the map of Fig. 12) which represent the 88.3% of the total wells in the same area. But on most of these wells, the depth construction and the quantity of withdrawals are unknown.

**Fig. 13:**
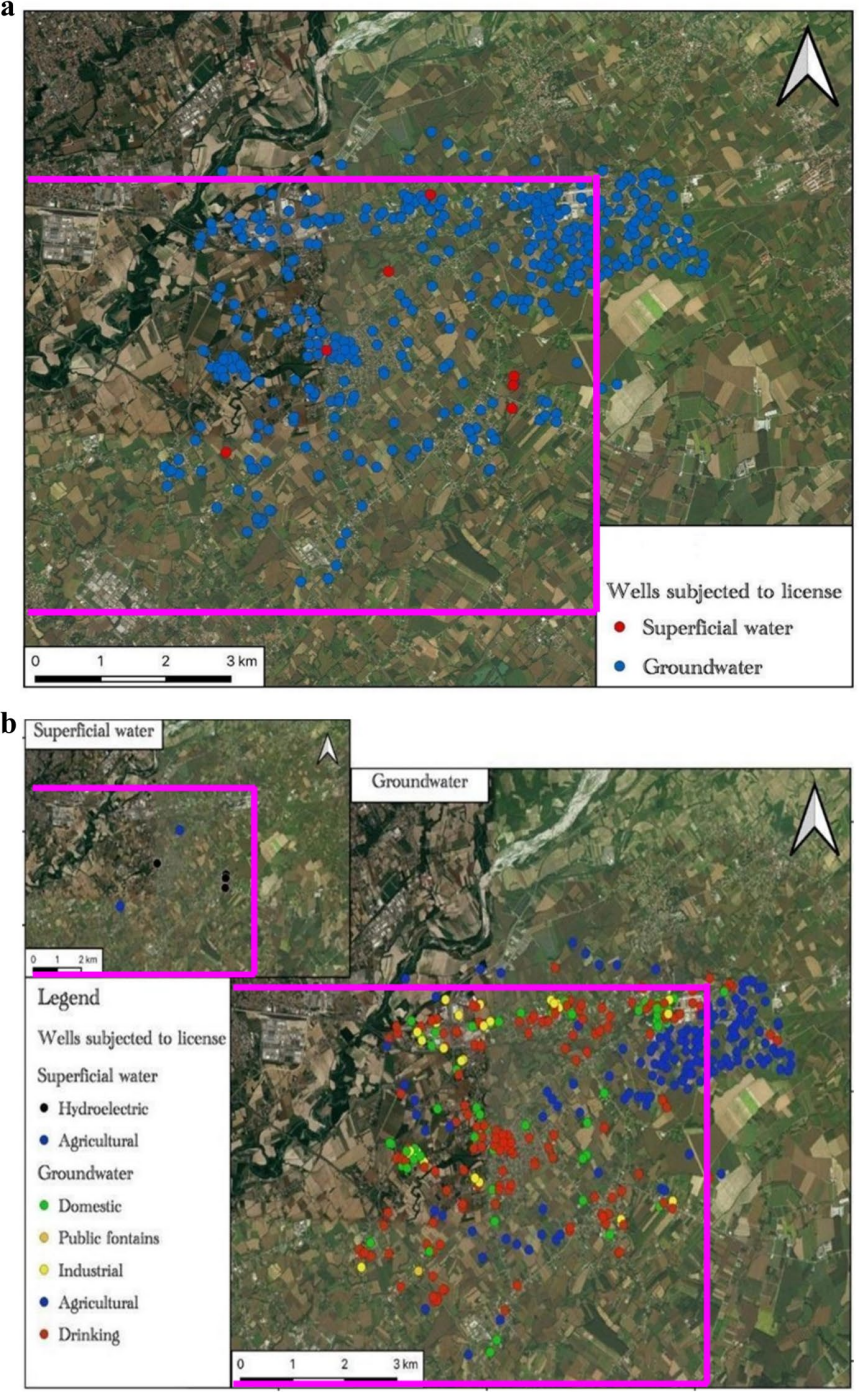
Map of the wells with the authorities to withdrawals: **a** map of the wells drawn from deep water (“groundwater” colored in blue) or superficial courses (colored in red); **b** map of the wells based on the type of use: from superficial water (top left), hydroelectrical activity (colored in black) and agricultural processes (colored in blue); from groundwater, domestic wells (colored in green), public fountain (colored in orange), industrial activity (colored in yellow), agricultural processes (colored in blue), and drinking water for potable uses (colored in red). The pink lines delimitate the Fiume Veneto sampling area of the research study

Figure 13, on the opposite, shows the map of the wells which have the authority to water withdrawals. Without it, Fig. 13a shows which and how many of these wells draw from deep water (colored in blue in the map of Fig. 13a) or superficial courses (colored in red in the map of Fig. 13a). The withdrawals on the superficial course are very low with respect to those on the deep water.

Regarding the superficial water wells (7 in total), these kinds of wells are used for hydroelectrical activity (95.6%) and less for agricultural processes (4.4%) (Fig. 13).

On the other side, agricultural processes (38.5%), drinking water for potable uses (39.5%), industrial activity (7.3%), and public fountains (0.3%) exploit wells from deep water aquifers (Fig. 13b).

Most of the authorized water wells are related to agricultural processes and potable uses. The domestic water wells represented in the maps of Fig. 13 are very low and underestimated compared to reality because they are not authorized, but only reported by the authorities. This confirms the lack of knowledge of how much water the domestic wells can drain and/or waste and above all what type of water they collect based on the aquifer in which they draw (superficial or intermediate and deep).

**Fig. 14:**
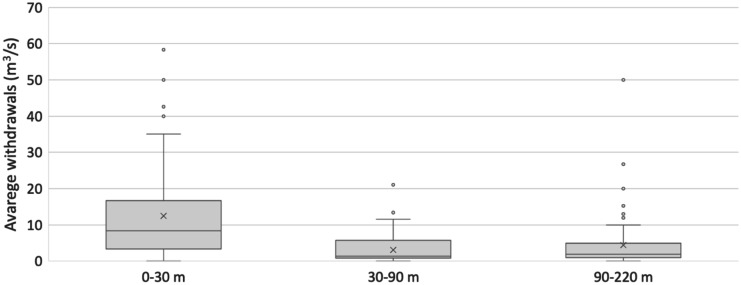
Average withdrawals in aquifer expressed in m^3^/s at different depths: 0–30 m, 30–90 m, and 90–220 m

Based on this data and information, licensed wells have been correlated with depth. Figure 14 shows the average withdrawals in the aquifer expressed in m^3^/s for the wells reported to the competent authorities and which have allowed them to be taken and which are built at different depths: 0–30 m (colored in blue—number of wells 182), 30–90 m (colored in orange—number of wells 76), and 90–220 m (colored in grey—number of wells 132). The graph in Fig. 14 shows that the maximum withdrawals are related to wells with depths less than 30 m (superficial water). Probably, since the activities declared for the withdrawal are mainly agricultural and industrial, it is cheaper to build a shallow well than a deep well.

However, it must be remembered that this calculation (the results of which are expressed in Fig. 14) was made considering only water wells with sampling licenses and not private ones, therefore most of the domestic wells, which characterize the Low Plain, are missing.

All wells considered for the calculation of the withdrawals are interested in all the aquifer systems present in the sampling area (A, B, and C in Fig. 14) and have continuous withdrawals seen that are naturally gushing wells and generally not equipped with discharge reducers. Accordingly, the withdrawals of all the wells considered (also for industrial and agricultural processes) are far higher than the real demands of the population (Zini et al., 2013).

Due to all these gaps in knowledge, it is not possible to accurately quantify the critical values of the over-exploitation of the aquifers, but they can be deduced by reasoning using the data provided by isotopic geochemistry.

Zini et al. (2011) stated that surface aquifers are critical due to high over-exploitation (mainly related to agricultural activities). However, the effective quantity of withdrawal, from surface, intermediate, and deep aquifers is underestimated because of the non-reporting of wells to the competent authorities present on the territory, especially water wells for domestic use. Just think that the estimate of the average withdrawals in the locality of Fiume Veneto was made based on approximately 350 wells declared with known withdrawals (mainly for agricultural activity) compared to the 2.107 wells declared to the authorities without knowing the average withdrawals. To these are added all the wells still not declared to the authorities. For this reason, it is not possible to calculate the correct amount of water withdrawn from these wells, which increases the criticality of the aquifers.

To be able to calculate this gap, it is necessary to take into account the waters of the superficial canals, into which the domestic wells that are not reported are discharged.

The data provided by the isotope analyses in this research work, in fact, indicate that the waters of the superficial canals are correlated with intermediate and deep aquifers. Taking into consideration that the domestic wells not reported are continuous flow, therefore, they withdraw water without any interruption, this suggests that not only the superficial aquifers are over-exploited, but also the intermediate and deep aquifers, which instead should be protected.

## Conclusions

Chemical and isotopic analyses carried out on water collected in wells, canal, and wastewater in the locality of Fiume Veneto (northeast of Italy) made it possible to discriminate water from different aquifers.

The tritium analyses, also, showed the presence of two main clusters representative of the age of the waters:

- Superficial aquifer (FVFR) with ^3^H values comparable to waters with recent recharge and values of δ^2^H and δ^18^O which reflect their meteoric nature
- A second larger group that includes intermediate and deep aquifer (FVAs and FVAd), wastewater (FVF), and superficial canal (C) waters with values of ^3^H classifiable as sub-modern waters (aged between 10 and 50 years) and which isotopic values suggesting the same origin and nature. Specifically, by correlating ^3^H with δ^18^O, the wastewater (FVF) and superficial canal waters (C) samples have the same isotopic footprint as intermediate and deep aquifer (FVAs and FVAd), both for enrichment in δ^18^O and for enrichment in SO_4_^2−^.

The analyses of ^3^H concentrations confirmed the hypotheses made with the previous geochemical and isotopic analyses, i.e., that the wastewater and the waters of the superficial canals have a similar nature to that of intermediate and deep aquifer water samples; therefore, it can be affirmed that the waters deriving from intermediate and deep aquifer wells, from the sewage system, and from the superficial canals that flow on the surface have the geochemical and isotopic composition of slow recharging deep waters.

This information is of fundamental interest because the studied area of Fiume Veneto is underestimated in the calculation of over-exploitation. Knowing, in fact, the type of water that flows into the surface channels into which the domestic wells are not declared to the competent authorities’ discharge, it is possible to identify the original aquifer from which most of the domestic wells draw and therefore understand the criticalities of the aquifers, both superficial than deep aquifer.

In the absence of reliable data, isotopic geochemistry is a useful tool to be able to fill this knowledge gap.

This is essential for the competent authorities to implement mitigation plans, especially in anticipation of water scarcity due to climate change.

## Data Availability

Not applicable.
